# Treatments in oral lichen planus: a review

**DOI:** 10.3389/froh.2026.1713845

**Published:** 2026-06-23

**Authors:** Ce Gou, Qinghua Zhang, Zhijun Zhang

**Affiliations:** 1 School of Clinical Medical, Chengdu Medical College, Chengdu, China; 2 Department of Stomatology, The First Afliated Hospital of Chengdu Medical College, Chengdu, China

**Keywords:** combination therapy, nonpharmacological treatment, oral lichen planus, pharmacological treatment, replacement therapy

## Abstract

Oral lichen planus is a chronic inflammatory disease of the oral mucosa characterized by pain and burning sensation. The etiology of OLP is complex and multifactorial, leading to recurrence and a lack of clear treatment methods. Drug therapy, including corticosteroids, calcineurin inhibitors, hydroxychloroquine, hyaluronic acid, and mTOR inhibitors, as well as non pharmacological treatments such as autologous platelet concentrates and photodynamic therapy, are the pillars of clinical management. This review is based on 46 articles published between 2020 and 2025, examining current treatment strategies and proposing innovative recommendations to strengthen existing treatments. This study provides new insights into drug combination strategies and non drug alternative treatment options for treating OLP, laying the foundation for future research and clinical trials aimed at developing more effective and personalized treatment plans.

## 1 Introduction

Oral lichen planus (OLP) is a chronic inflammatory disorder affecting the oral mucosa. It can result in pain, a burning sensation, dry mouth, and changes in taste perception for those affected. In more severe instances, it can lead to difficulties in speaking and have psychological implications (1). OLP is a disease of oral mucosa with a high incidence, affecting between 1% and 3.2% of the population (2). It tends to occur more often in middle-aged people, particularly among females, with a female-to-male ratio of 2:1 (3). Although the exact and definitive cause of oral lichen planus (OLP) remains somewhat elusive, earlier studies have suggested that OLP is likely a localized autoimmune disease, meaning that it arises from a malfunction or disorder within the body's immune system, specifically involving the dysfunction of T-cells. T-cells, which are a type of white blood cell, play a crucial role in the g immune response by identifying and attacking foreign pathogens. In the case of OLP, it is believed that these T-cells may mistakenly target the healthy tissues of the oral mucosa, leading to the characteristic inflammation and lesions associated with the condition (4, 5). The risk of malignant transformation from OLP to oral squamous cell carcinoma (OSCC) ranges from 1% to 2% across cases, with the erosive subtype posing the greatest risk (6), and the WHO has classified OLP as a potentially malignant condition (7, 8). Pharmacological treatments are widely recognized treatment methods and offer a wide range of options including topical steroids, topical calcineurin inhibitors, and hydroxychloroquine, among others (9). Non-pharmacological alternative therapies, including autologous platelet concentrates and photodynamic therapy, are increasingly being incorporated into clinical practice. In the present study, we tried to overview the treatments in Oral lichen planus, and explore emerging approaches to therapy. Datas were gathered from PubMed and Google Scholar. The search terms used included “Oral Lichen Planus”, “drug therapy”, “pharmacological treatments”, “non-drug therapy”, “nonpharmacological treatments”, “topical steroids/topical calcineurin inhibitors/topical retinoids”, and “platelet-rich plasma/platelet-rich fibrin/photodynamic therapy/photobiomodulation.” The articles reviewed were published between 2020 and 2025, with a total of 46 articles being assessed.

## 2 Oral lichen planus

### 2.1 Etiology of oral lichen planus

The precise cause of OLP remains unknown, and it is largely believed to be a multi-faceted condition triggered by various factors including genetic, immunological, and environmental elements (10).

#### 2.1.1 Psychological pressure

A significant number of LP patients have indicated that the initiation of their disease and the worsening of their lesions coincide with periods of heightened emotional stress (11). Individuals with OLP exhibit notably higher levels of stress, depression, and anxiety, which are linked to increased levels of cortisol in their saliva, as compared to healthy counterparts (12). This statistical correlation underscores the potential impact of psychological pressure on the development and progression of OLP.

#### 2.1.2 Genetic factors

Recent studies indicate that there might be a genetic tendency for OLP, as changes in genes related to the immune system are found in many OLP patients (13–15). These genetic elements have the potential to trigger irregular immune responses in the mouth lining, leading to OLP symptoms (16). Additionally, genetic polymorphism of vitamin D receptor is related to with the susceptibility to OLP in people of Han Chinese descent (17).

#### 2.1.3 Microbial imbalance

When the balance of gut bacteria is disturbed, it can weaken the gut lining, making it more permeable and allowing bacteria and their byproducts to enter the blood. This process could be a likely way to activate T cells, possibly leading to the start of OLP (18, 19).

#### 2.1.4 Hormone levels

Various investigations have identified that the changes in hormone levels is associated with the onset of OLP, especially in women who have gone through menopause. The endocrine system of women has substantial alterations during the process of menopause, significantly reducing the level of sex steroid hormones. On the other hand, increasing amounts of estrogen in the bloodstream of individuals with OLP have been associated with more severe manifestations of lesions (20).

#### 2.1.5 Drugs

Particular medications can play the role of haptens to cause an immune response, leading to instigate or worsen OLP lesions. These include beta-blockers, ACE inhibitors, nonsteroidal anti-inflammatory drugs, thiazide diuretics, antimalarials, and specific types of antibiotics (21). Lichenoid drug eruptions, also caused by drugs, clinically and histopathologically mimic lichen planus, but resolve completely upon drug withdrawal, which is different from the OLP (22).

### 2.2 Pathogenesis of oral lichen planus

The initiation of OLP is driven by the interplay between various immune cells, the cytokines and chemokines they release, and extracellular matrix proteins, collectively activating multiple pathogenic pathways (23).

In physiological status, keratinocytes discharge type IV collagen and laminin, emitting signals that inhibit apoptosis within the epithelial cells, which is essential for upholding the integrity of the basement membrane (24, 25). Oral mucosal injury activates T cells and induces apoptosis of keratinocytes (23). Activated CD8+ T cells infiltrate the epidermis and release tumor necrosis factor (TNF) and CD95L, which are associated with triggering cascade apoptosis of basal layer keratinocytes (26). Helper T cells (Th) 1 and Th2 jointly participate in the development of OLP, whose cytokines are expressed in saliva, serum, and peripheral blood mononuclear cells of OLP patients. Th1 activates cytotoxic CD8+ T cells to induce apoptosis of keratinocytes. In addition, interferon (IFN)-γ and IL-4 secreted by Th1 and Th2 cytokines play a role in regulating T cell differentiation and maintaining Th1/Th2 balance in physiological and pathological immune processes. IFN-γ participates in the maturation and activation of cytotoxic CD8+ T cells, induces the expression of adhesion factors in vascular endothelium, and the high expression of adhesion factors makes it easier for lymphocytes to enter human OLP tissue through the vascular wall, participating in the process of keratinocyte apoptosis (27).

**Figure 1:**
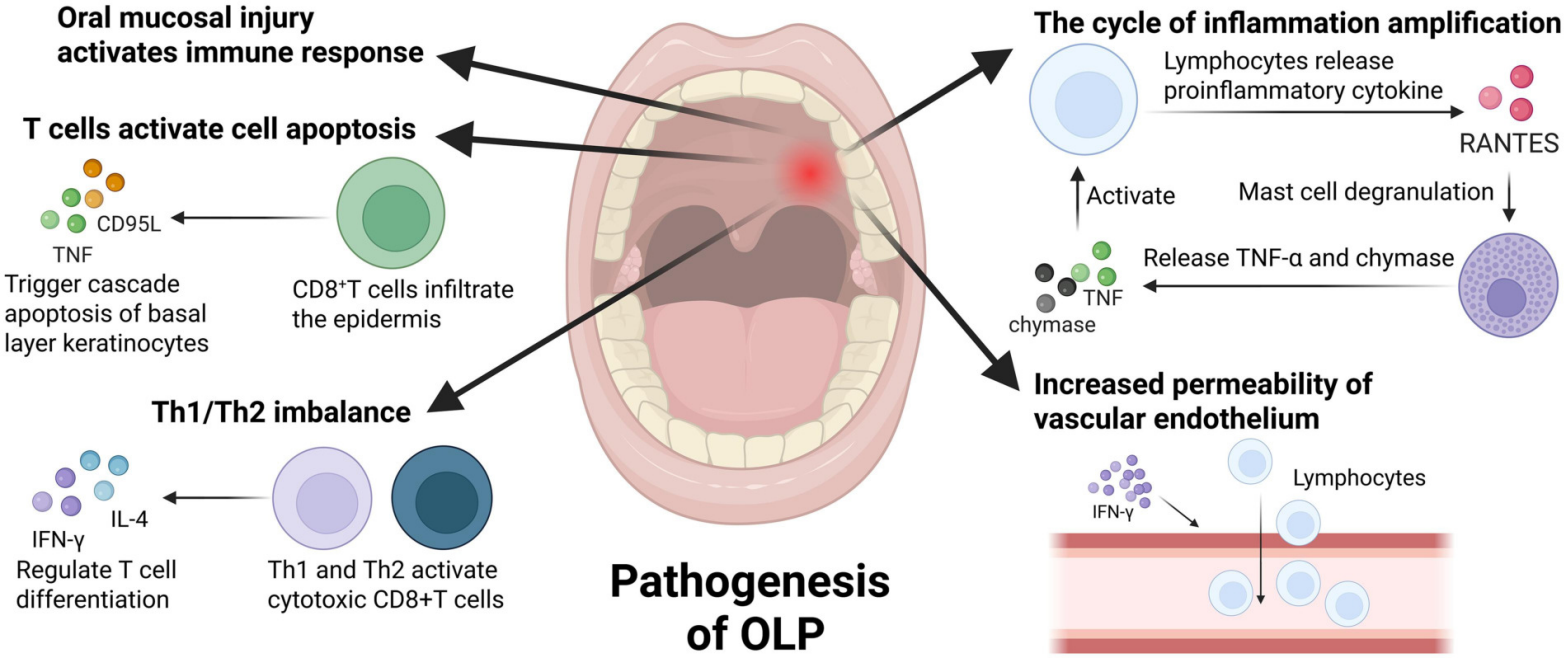
Pathogenesis of oral lichen planus.

On the other hand, activated lymphocytes and keratinocytes can release a proinflammatory cytokine RANTES, leading to the degranulation of mast cells, which then prompts the release of TNF-α and chymase. Lymphocytes are activated by TNF-α and discharge RANTES, initiating a vicious cycle which contributes to the prolonged nature of the disease (24, 25) (Figure 1).

### 2.3 Clinical presentation of oral lichen planus

OLP can be divided into six types based on clinical manifestations: reticular and papular, plaque-like, atrophic, erosive, and bullous (28).

#### 2.3.1 Reticular OLP and papular OLP

The reticular type of OLP, which is the most common, is marked by delicate white, lacy lines or papules, often on both sides of the the buccal mucosa, tongue, and gums (29). The papular type shows up as papules on the mouth lining, usually on the buccal mucosa, and often goes with the reticular type. Both types can occasionally bring about a slight ache or a sensation of burning (30).

#### 2.3.2 Plaque-like OLP

Plaque-like OLP is more common in smokers, featuring flat white spots on the inside of oral cavity. These lesions can show up as patches of different amounts, sizes and shapes, smooth or slightly raised (31). When there are inflammation, the lesions might have Wickham's striae around them (32). They commonly occur on buccal mucosa, tongue, and gingiva, may flake or crust while get irritated. This type can be without symptoms or cause a feeling of burning or itching (33).

#### 2.3.3 Erosive OLP

Individuals with Erosive OLP display painful ulcers, erosions, and erythematous areas in the oral cavity, typically located on the buccal mucosa, tongue and gingiva, appearring as “crater-like” (34). This type severely compromises eating and speaking, causes a feeling of burning, and enhances sensitivity of the lesions areas (35). To mitigate symptoms and prevent lesions deteriorated, early detection and personalized intervention are essential (36).

#### 2.3.4 Bullous OLP

Bullous OLP emerges as a unique yet uncommon clinical expression of OLP. This variant is characterized by fluid-filled vesicles or bullae in the oral cavity, frequently appearring on the buccal mucosa, tongue, and gingiva. These blisters are prone to rupture, causing painful ulcers (37). It's vital to have exact diagnosis and personalized management approaches for individuals with Bullous OLP to prevent its potential to worsen symptoms (38).

#### 2.3.5 Atrophic OLP

**Figure 2:**
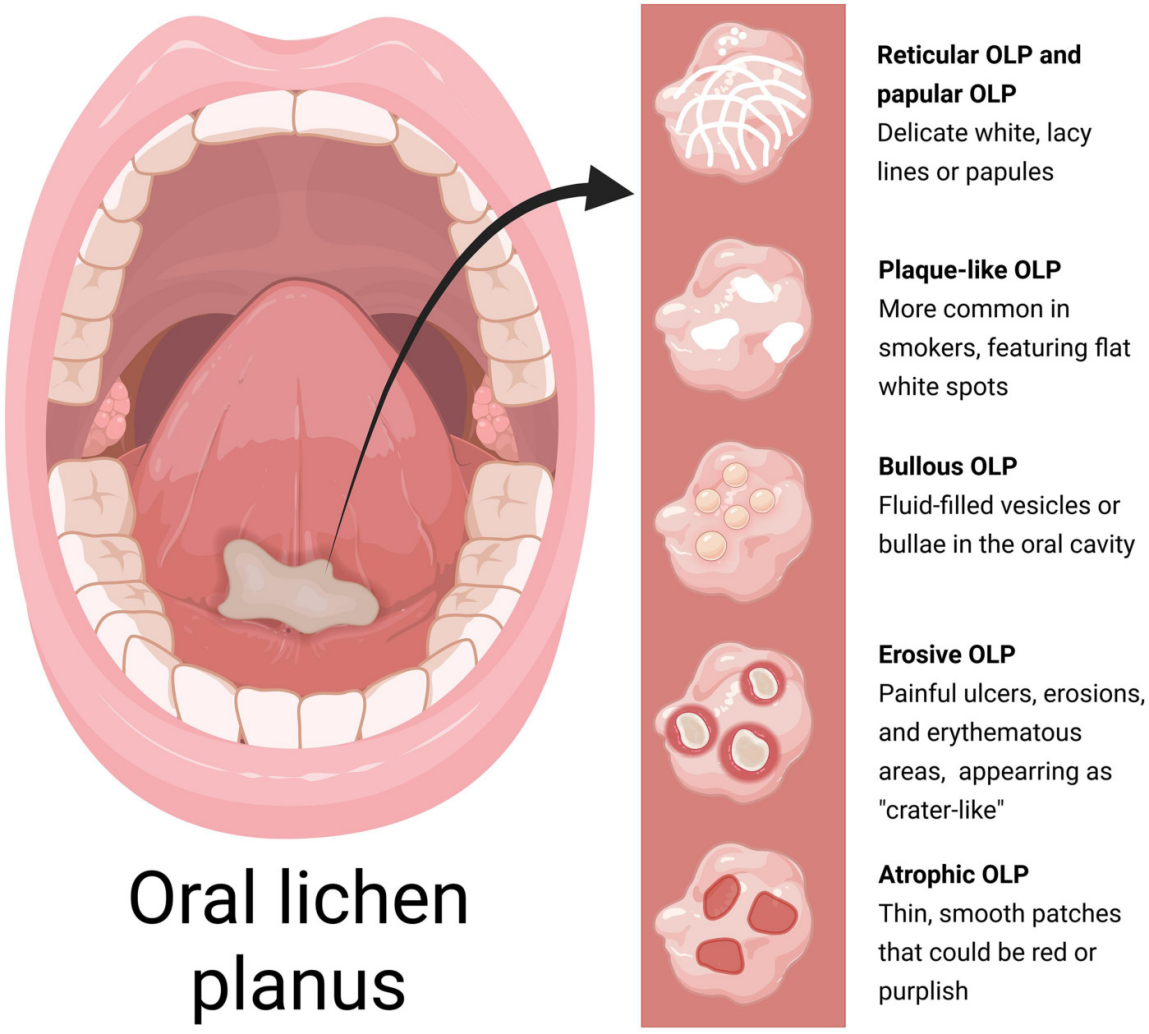
Clinical presentation of oral lichen planus.

Atrophic OLP exhibits thin, smooth patches that could be red or purplish on the oral mucosa, commonly found on the tongue (39). These lesions can also involve on diverse skin of the body, often accompanied by Wickham's striae. They can be symptom-free or lead to burning or itching sensations (Figure 2).

### 2.4 Scoring systems

To effectively assess and monitor the progression of oral lichen planus (OLP) and to provide a standardized approach to treatment planning and evaluation, several scoring systems have been meticulously developed. These systems meticulously evaluate OLP's severity, considering clinical indicators like lesion extent, patient-reported symptoms such as pain intensity and taste alterations, and the condition's impact on speech and daily life. They assist in tracking disease changes, guiding treatment decisions, and enabling comparisons across studies, enhancing OLP management and research (40) (Table 1).

**Table 1:** Scoring systems of oral lichen planus.

Scoring systems	Evaluate aspects	Reference study
Oral Health Impact Profile (OHIP-14)	Oral health-related quality of life	Slade (41)
Visual analog scale (VAS)	Oral pain	Saberi (42)
Reticulation/keratosis, erythema, and ulceration (REU) scores	Disease severity	Park (43)
Oral Disease Severity Score (ODSS)	Disease severity	Ormond (44)
Numerical rating scale (NRS)	Oral pain	Park (43)
Thongprasom score	Symptoms, severity of lesions	Selaru (45)
Self-administered 14-item Hamilton anxiety and depression scale (HAD)	Anxiety and depression	Salinas-Gilaber (46)
White-Erosive-Atrophic (WEA)	Disease severity	Gobbo (40)
Autoimmune Bullous Skin Disorder Intensity Scores (ABSIS)	Clinical parameters	Bender (47)

## 3 Pharmacological treatments

### 3.1 Corticosteroids

Glucocorticoids play a crucial role in the growth and development, while corticosteroids, as synthetic counterparts of glucocorticoids, exhibit properties of anti-inflammatory and immunosuppressive (48). Topical steroids remain the most cost-effective and efficient option of OLP treatments currently, such as triamcinolone acetonide and clobetasol, while systemic medication usually chooses prednisone (3, 49).

#### 3.1.1 Triamcinolone acetonide

Triamcinolone acetonide, a versatile and widely utilized medication belonging to the class of corticosteroids, is available in a diverse range of pharmaceutical forms to cater to different medical needs and modes of application, including ointments, gels, mouthwashes, sprays, and pastes, which is most frequently medication employed for the treatment of mucosal lesions (50).

Triamcinolone acetonide can reduce the expression of IL-9 in the lesions, leading to the decline of MMP9, to relieve the symptoms of OLP (51). In clinical settings, the prevalent forms of triamcinolone acetonide are the 0.1% ointment and 0.1% mouth rinse (52–55). However, the intralesional injection of triamcinolone (40 mg/mL) results in fewer adverse effects compared to the use of triamcinolone mouth rinse (56, 57). Besides, aministration of diclofenac sodium sustained-release tablets has been shown to provide relief from allergic reactions induced by triamcinolone acetonide injections (58).

Specific patient populations, particularly those grappling with persistent or intricate medical conditions, might require the continuation of this medication's use over an extended period. There arises a possibility that the lesions may develop resistance to the medication's effects as time progresses or produce certain complication. In such scenarios, a strategic transition to combination therapy becomes imperative. Treating with a combination of triamcinolone acetonide and herbal medication offers antifungal benefits and helps avoid candidiasis, a frequent side effect of corticosteroid use (59). Moreover, the application of 1% nanocurcumin alongside 0.1% triamcinolone acetonide can notably boost the healing of lesions (60). In addition, utilizing HA combined with triamcinolone for OLP lesions leads to a reduced rate of symptom reappearance (61, 62).

#### 3.1.2 Clobetasol

Clobetasol is a highly potent form of topical corticosteroid that stands out among other similar medications due to its exceptional efficacy. It exerts its powerful anti-inflammatory effect by effectively preventing various inflammatory processes within the body. These processes include edema, which is the swelling of tissues due to the accumulation of fluid; fibrin deposition, a process where fibrin, a protein involved in blood clotting, is deposited in tissues, contributing to inflammation; vasodilation, the widening of blood vessels which can exacerbate inflammation; and phagocytic activity, the process by which certain cells engulf and destroy pathogens and damaged cells, which can be part of the inflammatory response (63).

Studies find that clobetasol propionate can reduce the level of IL-1β in saliva, indicating that IL-1β plays a crucial role in the therapeutic mechanism of clobetasol in OLP (64). The observed increase in epithelium and the corresponding decrease in lamina propria can be elucidated as a direct result of clobetasol's ability to facilitate the healing of epithelial tissues and to mitigate inflammation at the interface (65). The effectiveness of Clobetasol in treating conditions that require anti-inflammatory intervention is underscored by the significant remission rates observed in patients. Specifically, a remarkable 56–75% of patients who are treated with Clobetasol experience complete remission from their symptoms, indicating a high level of success in resolving the underlying inflammatory issues. In contrast, when patients are treated with other corticosteroids, the percentage of those achieving complete remission drops significantly, ranging from 30 to 15% (66, 67). In the realm of clinical practice, the most prevalent and widely utilized form of clobetasol is the 0.05% Clobetasol Propionate ointment (52, 68–72).

However, the application of corticosteroids, including Clobetasol, is not without its drawbacks, as it can give rise to a host of adverse effects that patients and healthcare providers need to be vigilant about. One of the common side effects is oral candidiasis, a fungal infection that not only affects the patient's oral health but can also lead to difficulties in eating and speaking. Additionally, patients may experience hypogeusia, further impacting their quality of life and enjoyment of food (73). Moreover, the prolonged use of topical Clobetasol gel poses additional risks, particularly the potential for adrenal suppression (74). Combining the potent corticosteroid clobetasol with the antifungal agent 2% miconazole serves as a protective strategy against iatrogenic candidosis, mitigating the immunosuppressive side effects of topical steroids (75).

#### 3.1.3 Prednisone

Prednisone, a corticosteroid medication, may alleviate OLP by exerting its anti-inflammatory and immunosuppressive effects on the immune system. When encountering refractory and severe multifocal presentations in patients, the medication prednisone is often utilized, with the route of administration being oral intake (76). Specifically, prednisone works by reducing the production and release of pro-inflammatory cytokines, such as TNF-α and IL-6, contributing to dampen the immune response and alleviate the inflammation and tissue damage associated with OLP (77). However, it is important to note that prolonged use of prednisone, due to its immunosuppressive effects, may increase the risk of developing oral candidiasis infection, a common complication that requires careful monitoring and management in patients undergoing long-term prednisone therapy (Table 2).

**Table 2:** Corticosteroids in oral lichen planus.

Drug	Reference study	Indication	Intervention	No. of pts	Duration	Frequency	Outcome measure
Triamcinolone acetonide	Zhao (57)	erosive non-gingival OLP	0.5 mL, 10 mg TA	25	2 wks	once a week	erosion size; NRS; PI; CPI
Triamcinolone acetonide	AlMutairi (78)	OLP	0.1% TA	40	4 wks		Erosive area; VAS
Triamcinolone acetonide with tacrolimus	Walia (79)	OLP	0.5 mL TA (40 mg/mL)	56	8 wks	once a week	VAS; Thongprasom score
Triamcinolone acetonide	Riyaz (52)	OLP	0.1% TA	40	15 days		VAS
Triamcinolone acetonide	Bajoria (53)	OLP	0.1% TA	50			burning sensation; severity of lesion; side effects
Triamcinolone acetonide	Kaur (54)	OLP	0.1% TA	40	15 days		ulcerative lesion type; erosive area; VAS
Triamcinolone acetonide with oral acitretin	Vinay (55)	OLP	0.1% TA oral paste+25 mg of oral acitretin	32	28 wks	acitretin once a day;TAC oral paste thrice daily	ODSS; VAS; OHIP-14
Triamcinolone acetonide mouth rinse with nanocurcumin gel	Bakhshi (60)	erosive or ulcerative OLP	1% nanocurcumin gel and 0.1% TA mouth rinse	17	4 wks	thrice a day	REU; clinical score
Triamcinolone with HA	Agha-Hosseini (61)	OLP	7 mg HA powder was dissolved in 40 mg/mL TA 1 mL for each 2 cm^2^	14			VAS
Triamcinolone with HA	Rodriguez-Galvez (62)	OLP	TA 0.2% + HA 0.1% in orabase	20	4 wks	twice daily	Thongprasom scale; OHIP-14; VAS; SOD; GSH
Clobetasol propionate	Riyaz (52)	OLP	0.05% clobetasol propionate	40	15 days		VAS
Clobetasol propionate	Vaidya (68)	OLP	0.05% clobetasol propionate ointment		8 wks	twice daily	Site score; Burning Sensation score; REU
Clobetasol propionate	Kamal (69)	OLP	one capsule of probiotics complex obtained from Biovea + clobetasol propionate 0.05% in orabase gel	30	4 wks	twice daily	Thongprasom criteria; numerical rating scale; candidal load
Clobetasol propionate	Santonocito (70)	symptomatic OLP	clobetasol propionate 0.05%	20	12 wks	twice daily;	NRS; Thongprasom score
Clobetasol propionate	Mamadapur (72)	OLP	Clobetasol Propionate ointment 0.05%	30	4 wks	twice daily	NPS
Clobetasol propionate patches	Brennan (73)	OLP	Rivelin®-CLO patches	69	4 wks		CGI; ODSS
Prednisone	Jiang (77)	erosive OLP	oral prednisone acetate at a dose of 0.4 mg/(kg·d)	30	1 wks		erosion size; NRS
Prednisone	Wang (76)	severe erosive OLP	prednisone (0.4 mg/kg/d)	31	4 wks		erosion size; NRS; Thongprasom score

### 3.2 Calcineurin inhibitors

Calcineurin inhibitors are a class of immunomodulatory agents that play a crucial role in the regulation of immune responses by targeting specific intracellular pathways within T-lymphocytes. These inhibitors work by binding to distinct intracytoplasmic proteins, which are critical components in the signaling pathways responsible for T-cell activation and proliferation. This binding event initiates a cascade of molecular interactions that ultimately lead to the inhibition of calcineurin, preventing the transcription and production of various cytokines essential for immune response, which makes them valuable tools in managing immune-mediated diseases (80).

#### 3.2.1 Tacrolimus

**Figure 3:**
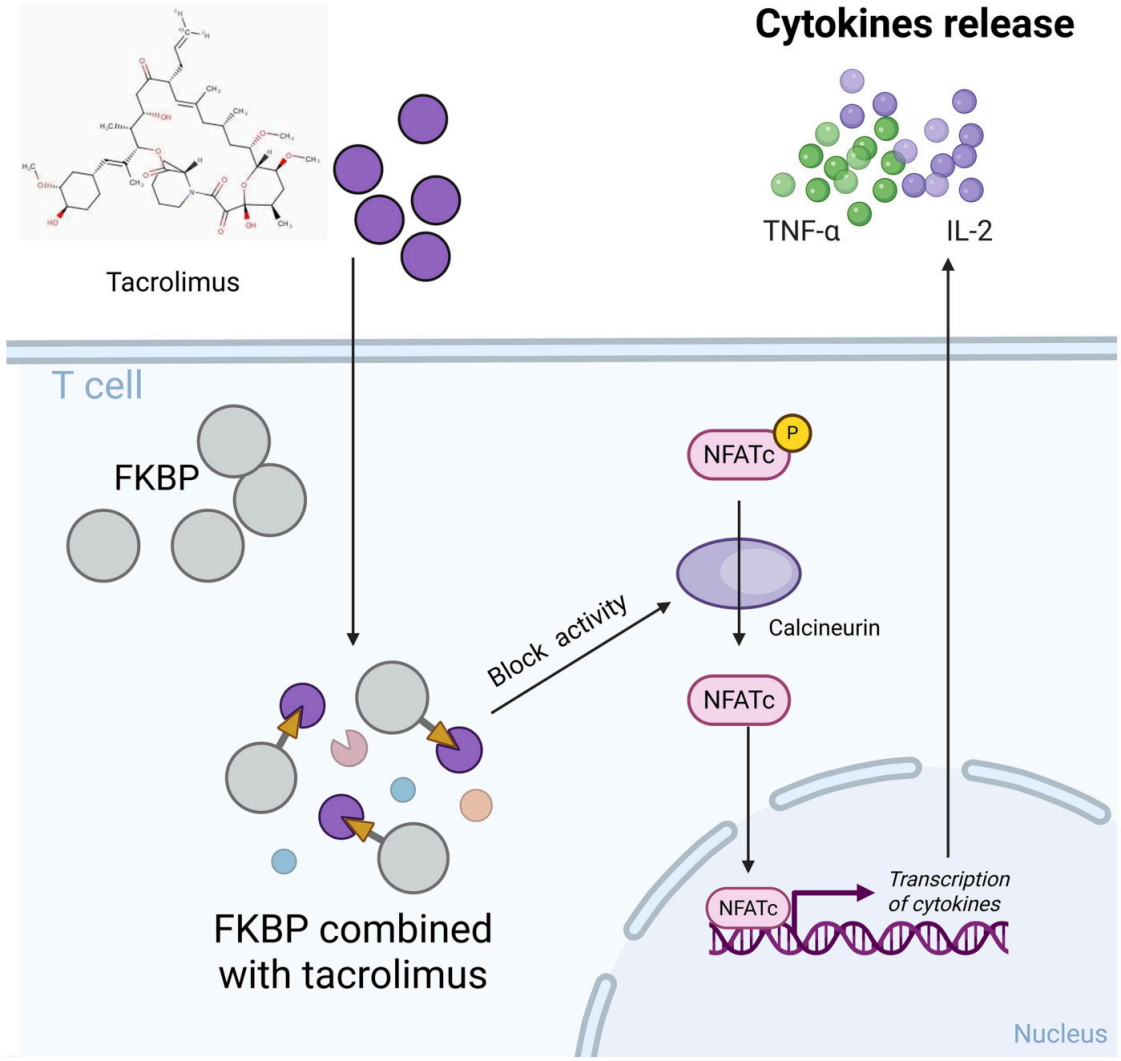
The mechanism of tacrolimus in treating oral lichen planus.

Tacrolimus is a potent immunomodulator that plays a crucial role in suppressing T-cell activation. Specifically, tacrolimus effectively blocks calcineurin's activity by binding to the cytosolic FK-binding protein (FKBP), thereby preventing the pro-inflammatory cytokines such as interleukin-2 (IL-2) and tumor necrosis factor-alpha (TNF-α) from being produce (81) (Figure 3). Additionally, Tacrolimus can decrease the proliferation of regulatory T cells (Tregs) and inhibit the NF-κB pathway, a signaling cascade involved in the pathogenesis of OLP (82, 83). Tacrolimus was shown to activate the TGF-βRI/Smad3 pathway, leading to increased transcription and production of IL-37 within gingival epithelial cells. This upregulation of IL-37 is particularly important in mitigating inflammatory conditions affecting the oral mucosa of OLP (84). Furthermore, Tacrolimus has been shown to reduce the expression of caspase-3, a critical enzyme in the apoptosis-inducing protease pathway associated with keratinocyte apoptosis. In the context of OLP lesions, this reduction in caspase-3 expression indicates impaired T-cell viability and reduced local inflammation. This finding explains Tacrolimus not only suppresses immune responses but also protects epithelial cells from apoptosis, thereby contributing to the healing process and symptom relief in OLP patients (85, 86).

Topical immunosuppressive tacrolimus is a viable option for OLP patients who do not exhibit a response to corticosteroid treatment, particularly in cases of refractory erosive oral lichen planus (71, 87). In clinical practice, the concentration of tacrolimus ointment typically ranges from 0.03% to 0.1%. The lower concentration (0.03%) is often used for milder cases or more sensitive areas (78), while the higher concentration (0.1%) may be preferred for more severe or resistant cases of EOLP (88).

Combination therapy involving tacrolimus is generally more effective than monotherapy in treating OLP (79). Platelet-rich plasma (PRP) gel combined with tacrolimus proves superior to tacrolimus alone for EOLP treatment. Adjunctive PRP gel significantly enhances the efficacy of tacrolimus ointment in treating erosive oral lichen planus (EOLP), effectively reducing lesions, alleviating pain, and improving the oral immune microenvironment with a lower recurrence rate (89). Despite its effectiveness, topical tacrolimus does come with potential side effects. One notable side effect is the transient burning or painful sensation experienced by some patients upon application. This discomfort is believed to result from the release of neuropeptides such as substance P, which stimulate sensory neurons in the affected area (90).

#### 3.2.2 Cyclosporine

In the realm of clinical practice, a topical cyclosporine oral solution with a concentration of 100 mg/mL is frequently employed for the management of oral lichen planus (91, 92). Patients who have not responded adequately to steroid treatments experience a notable reduction in pain and a decrease in the extent of their disease during therapy with a low-dose cyclosporine mouth rinse (90). However, it is important to note that topical cyclosporine is not typically considered the first choice when alternative therapeutic options to topical steroids are required. This reluctance to use cyclosporine as the initial alternative may be attributed to several factors, including its potential side effects and the limited, often conflicting, data available regarding its efficacy (93, 94).

One of the more concerning side effects of cyclosporine is gingival enlargement and hypertrophy, which can occur when patients are treated with high dosages of the medication, leading to relevantly high serum levels of cyclosporine (95). This condition can give rise to pseudo pockets, which are spaces between the teeth and gums that are not naturally present and can harbor bacteria. The accumulation of bacteria in these pockets can lead to gingivitis, an inflammation of the gums, and if left untreated, can progress to periodontitis, a more severe form of gum disease that affects the tissues and bone supporting the teeth. In the most severe cases, periodontitis can result in tooth loss, compromising oral health and function (96). These potential side effects necessitate careful monitoring of patients receiving cyclosporine treatment and a cautious approach to prescribing higher doses, particularly over extended periods.

### 3.3 Hydroxychloroquine

Up until now, the treatment of oral lichen planus (OLP) has primarily focused on palliative care, with the best outcomes achieved through the application of topical or systemic corticosteroids. Nevertheless, the increasing incidence of secondary candidiasis and the development of treatment-resistant forms of OLP have spurred the search for innovative therapeutic agents and strategies to combat this issue (97). Hydroxychloroquine (HCQS), an anti-malarial drug, emerged as a potential treatment option. In 1993, it was first used as a standalone therapy for OLP and reported positive results, particularly in diminishing erosive lesions and relieving pain. Extensive documentation supports the efficacy of hydroxychloroquine in ameliorating erosive oral lichen planus, mitigating associated pain, and reducing erythema (71, 76, 98, 99).

Hydroxychloroquine exerts its immunomodulatory effects by downregulating the formation of major histocompatibility complex (MHC) class II-peptide complexes. This intricate process involves the reduction of antigen presentation on the surface of antigen-presenting cells, thereby impeding the activation of CD41T cells. By diminishing the activation of these key immune cells, hydroxychloroquine effectively curtails the overall immune response, which may be the fundamental mechanism driving its therapeutic efficacy in the treatment of oral lichen planus. Moreover, hydroxychloroquine interferes with toll-like receptor (TLR) signaling pathways, a critical component of the innate immune system. This interference leads to a decrease in the production of proinflammatory cytokines, such as interleukin-1β(IL-1β), tumor necrosis factor-α(TNF-α), and interleukin-6 (IL-6), which are pivotal in initiating and sustaining inflammatory processes. By mitigating the production of these proinflammatory cytokines, hydroxychloroquine exerts a potent anti-inflammatory action, making it a promising treatment option for managing the inflammatory aspects of OLP (100). This dual mechanism of action, targeting both adaptive and innate immune responses, underscores the potential of hydroxychloroquine as a valuable therapeutic agent in the management of oral lichen planus, offering a multifaceted approach to modulate the immune dysregulation that characterizes this condition.

A daily regimen of 400 milligrams of HCQS administered as monotherapy over a six-month period has emerged as a potentially promising curative approach for patients suffering from OLP. This treatment strategy has demonstrated encouraging results, as evidenced by the lack of significant adverse outcomes and the absence of reported recurrences of oral lesions during the subsequent one-year follow-up period (101). This finding underscores the potential efficacy and safety of this therapeutic regimen in the management of OLP.

### 3.4 Hyaluronic acid

Hyaluronic acid (HA), a glycosaminoglycan, has been shown to stimulate angiogenesis, reduce exudation, provide vasoprotection, and exhibit fibrogenic effects in inflamed tissues that exhibit impaired healing processes (102). Topical application of 0.2% hyaluronic acid orabase has demonstrated efficacy in the treatment of OLP (102, 103). In clinical practice, HA is frequently employed in combination with other therapeutic agents for the management of OLP, given its potential synergistic effects (61, 62, 70). Despite the promising biological activities of HA, the available data regarding its use in OLP are limited and often contradictory. This lack of consensus in the literature can be attributed to the presence of various methodological biases in the existing studies on HA, including but not limited to, small sample sizes, suboptimal study designs, inconsistent methodologies, and disparate outcome measurement criteria (102–104). These biases may significantly impact the validity and generalizability of the study findings. Future research should aim to address these methodological shortcomings and explore the optimal dosage, formulation, and duration of HA treatment for OLP, as well as its potential interactions with other therapeutic agents.

### 3.5 mTOR inhibitor

Rapamycin, a macrolide immunosuppressant, is employed in the treatment of refractory chronic erosive oral lichen planus (CEOLP), a severe form of the disease characterized by painful oral ulcers. Rapamycin attaches to the immunophilin FK506 binding protein 12 (FKBP12), thus preventing the activation of the mammalian target of rapamycin (mTOR) complex. This inhibition of mTOR signaling results in immunosuppressive activity, impairing T-cell activation and proliferation, and exerting antiproliferative and anti-angiogenic effects (104).

In clinical practice, a concentration of 1 mg/mL of rapamycin is commonly used in the treatment of CEOLP (105). Although rapamycin has been associated with some side effects, such as increased risk of infection and bone marrow suppression, its systemic side effects are relatively minimal due to the limited absorption of the drug into the bloodstream (106). This reduced side effects make rapamycin a potentially attractive therapeutic option for the management of CEOLP (Table 3).

**Table 3:** Other drugs in oral lichen planus.

Drug	Reference study	Indication	Intervention	No. of pts	Duration	Frequency	Outcome measure
Tacrolimus mucoadhesive patches	Ibrahim (86)	erosive/atrophic OLP	TAC mucoadhesive patches (10%)	10	8 wks	twice daily	CS; TAA; VAS; punch biopsy
Tacrolimus	AlMutairi (78)	OLP	0.03% TAC	40	4 wks		Erosive area; VAS
Tacrolimus	Kiyani (88)	erosive OLP	0.1% TAC	12	12 months	thrice a day	WEA-MOD
Tacrolimus + PRP	Huang (106)	erosive OLP	TAC + PRP				lesion area scores; VAS
Tacrolimus orabase	Schroeder (71)	symptomatic OLP	0.1% TAC orabase	12	30 days	4 times a day	ODSS; VAS; OHIP-14; Beck Anxiety Scale; Hedonic Scale
Cyclosporine mouth rinse	Monshi (90)	OLP (patients recalcitrant to topical steroids)	2 mL CsA (100 mg/mL)	21	4 wks	thrice a day	histology; immunofluorescence; ELISA; VAS; PGA; DLQ
Cyclosporine oral solution	Georgaki (91)	symptomatic OLP	CsA oral solution 100 mg/mL	16	4 wks	thrice a day	cytologic smear; VAS; Thongprasom score
Enteral hydroxychloroquine	Raj (100)	active erosive OLP	oral 200 mg of HCQ	45	6 months	twice daily	HCQS; REU; Tel Aviv-San Francisco Scale; VAS; BURN
Hydroxychloroquine	Wang (76)	severe erosive OLP	HCQ	31	4 wks		lesion area scores; Thongprasom; NRS
Hydroxychloroquine	Chirravur (98)	symptomatic OLP	HCQ 200 mg	36		twice daily	Pain; reticulation; erythema; ulceration scores
Hydroxychloroquine	Schroeder (71)	atrophic/erosive/ulcerative OLP	0.1 g HCQ	50	4 wks	twice daily	RHU; VAS
Hydroxychloroquine	Wang (76)	severe erosive OLP	HCQ (5 mg/kg/d)	31	4 wks		erosion size; NRS; Thongprasom score
Hyaluronic acid was mixed with triamcinolone	Agha-Hosseini (61)	OLP	7 mg HA powder was dissolved in 40 mg/mL TA, 1 mL for each 2 cm^2^	14			VAS
Hyaluronic acid was mixed with triamcinolone	Rodriguez-Galvez (62)	OLP	TA 0.2% + HA 0.1% in orabase	20	4 wks	twice daily	Thongprasom scale; OHIP-14; VAS; SOD; GSH
Hyaluronic acid orabase	Shetty (101)	OLP	0.2% HA orabase	25	2 wks	thrice a day	erosion size; VAS
Anti-inflammatory mouthwash	Santonocito (70)	symptomatic OLP	10–20 mL anti-inflammatory mouthwash (HA 0.3%)	20	12 wks	thrice a day	NRS score; Thongprasom score
Hyaluronic acid preparation	Hashem (102)		0.2% HA	20	4 wks	thrice a day	erosion size; VAS
Rapamycin	Soria (105)	recalcitrant CEOLP	topical rapamycin (1 mg/mL)	7	12 wks	twice daily	blood absorption of topical rapamycin; erosion size
Rapamycin	Samimi (104)	erosive OLP	rapamycin solution (1 mg/mL)	39	12 wks	twice daily	recurrence

## 4 Nonpharmacological treatment

### 4.1 Autologous platelet concentrates

Autologous platelet concentrates (APCs), which include Platelet-rich plasma (PRP) and Platelet-rich fibrin (PRF), have emerged as innovative and effective injectable therapies in the field of dermatology, particularly in the management of lichen planopilaris cases that have proven resistant to traditional treatment modalities such as corticosteroids (107, 108).

#### 4.1.1 Platelet-rich plasma

Platelet-rich plasma (PRP) is essentially a concentrated form of plasma derived from the patient's own blood, serving as a valuable alternative source of growth factors for a wide array of dental procedures and oral mucosal lesions. Prepared through centrifugation of whole blood, PRP boasts a significantly higher concentration of platelets compared to normal plasma—typically 3–5 times the baseline levels. This elevated platelet count translates to an intensified reservoir of growth factors, which are critical for tissue repair and regeneration. Alongside this enhanced platelet density, PRP also contains increased levels of coagulation factors, such as fibrinogen and thrombin, which facilitate hemostasis and provide a structural matrix for cell adhesion and migration (109).

When platelets within the PRP are activated via physiological stimuli (e.g., calcium chloride or thrombin), they release a diverse array of growth factors, including platelet-derived growth factor (PDGF), transforming growth factor-beta (TGF-β), vascular endothelial growth factor (VEGF), and fibroblast growth factor (FGF). These growth factors orchestrate a cascade of biological events pivotal for healing, such as stimulating cell proliferation and differentiation, promoting neoangiogenesis to enhance blood supply, detoxifying the wound microenvironment by neutralizing inflammatory toxins, and driving cellular regeneration through recruitment of stem cells and epithelialization (110).

By facilitating these regenerative processes, PRP not only accelerates wound healing in dental surgeries (e.g., periodontal defects, bone grafting) and oral lesions (e.g., mucositis, ulcers) but also mitigates associated morbidity. Its inherent anti-inflammatory action, mediated by factors like interleukin-10 (IL-10) and downregulation of pro-inflammatory cytokines, reduces postoperative pain, swelling, and infection risk (111). This dual role of promoting tissue restoration while modulating inflammation underscores PRP's therapeutic potential in optimizing clinical outcomes and patient recovery in oral medicine and dentistry.

#### 4.1.2 Platelet-rich fibrin

Platelet-rich fibrin (PRF) represents a cutting-edge advancement in regenerative medicine, distinguished by its intricate three-dimensional fibrin network structure. This unique architecture not only accelerates wound healing but also enhances the body's immune response and fosters neovascularization—the formation of new blood vessels—critical for restoring tissue perfusion and oxygenation. PRF's composition is particularly noteworthy as it contains host immune defense cells, specifically leukocytes, which are integral components of the body's innate and adaptive immune systems. These leukocytes, along with platelets and growth factors, play a pivotal role in the three key stages of wound healing that PRF actively promotes.

**Figure 4:**
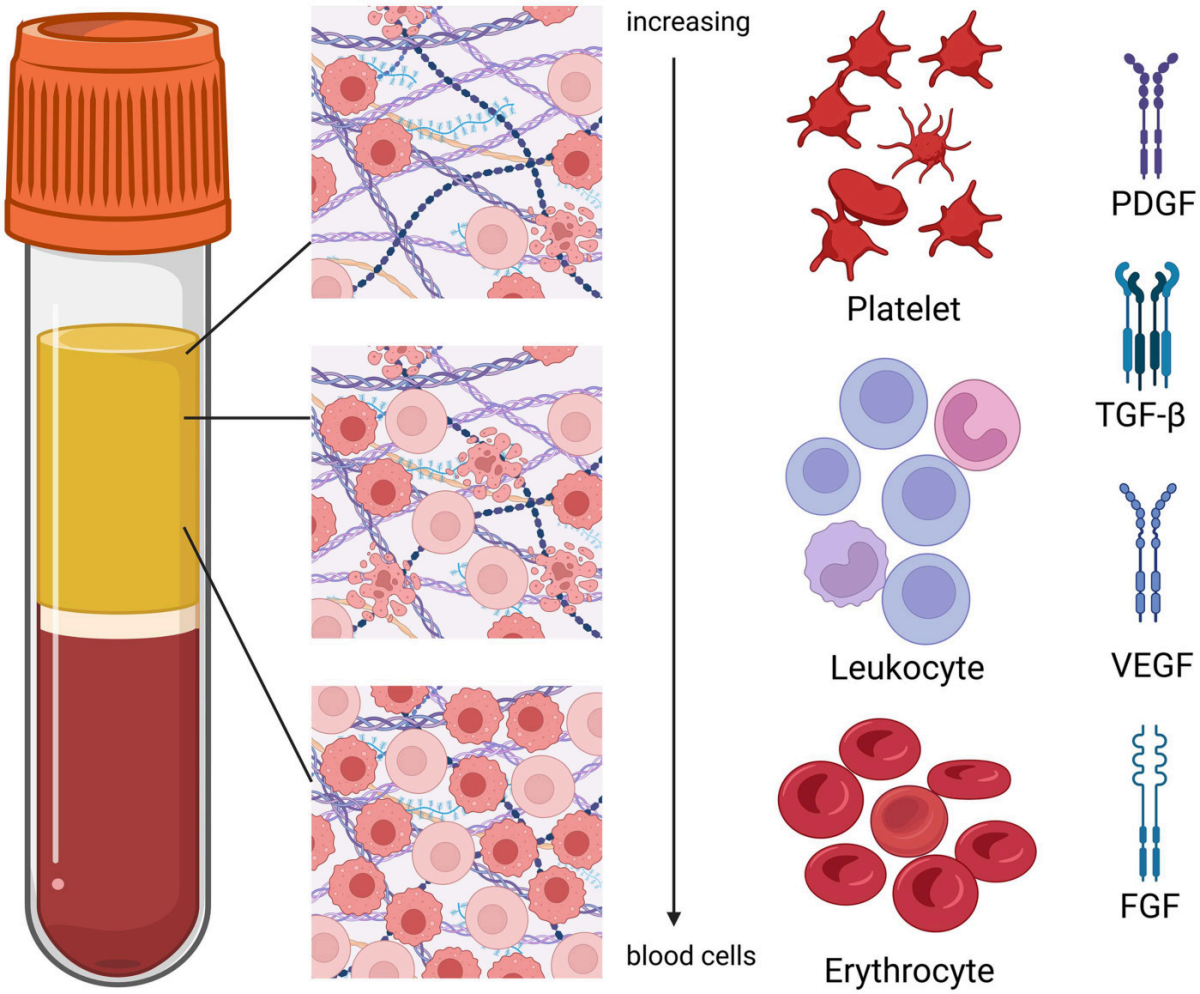
Three-dimensional fibrin network structure of platelet-rich fibrin.

Firstly, PRF facilitates angiogenesis by releasing growth factors such as vascular endothelial growth factor (VEGF) and fibroblast growth factor (FGF). These factors stimulate the proliferation of endothelial cells, which form new blood vessels, ensuring the wounded area receives essential nutrients and oxygen for tissue repair and regeneration. Secondly, it enhances the immune response through the activity of leukocytes, including neutrophils and macrophages, which combat infections, phagocytose pathogens, and regulate inflammatory processes. This dual action reduces the risk of wound complications and promotes a balanced healing environment. Lastly, PRF promotes epithelial proliferation by supporting the migration and differentiation of keratinocytes, the primary cells of the skin's outermost layer. This regeneration restores the skin's integrity and function, leading to faster closure of wounds and reduced scarring (Figure 4).

Furthermore, PRF's fibrin matrix acts as a physical scaffold, retaining growth factors and immune cells for prolonged release, thereby sustaining therapeutic effects over time. This natural, autologous material avoids the risks associated with exogenous additives, making it a safe and effective modality for enhancing tissue repair in various clinical settings, including dental, orthopedic, and dermatological applications (112).

Intralesional administration of Platelet Rich Plasma (PRP) and Platelet-rich fibrin (PRF) has demonstrated remarkable efficacy in the treatment of oral lichen planus (OLP), offering distinct clinical advantages over conventional therapies. This innovative approach leverages the regenerative potential of autologous platelet concentrates, which are rich in growth factors, cytokines, and immune-modulating components. When injected directly into OLP lesions, PRP and PRF not only accelerate tissue repair and reduce inflammation but also exhibit significantly lower recurrence rates compared to traditional treatments such as topical corticosteroids or systemic immunosuppressants (113–118).

### 4.2 Phototherapy

Phototherapy approaches in oral lichen planus treatment encompass a diverse array of modalities, historically including UVA- and UVB-based light therapies—methods that, while once prevalent, are now largely phased out due to significant safety concerns. Contemporary phototherapeutic strategies have shifted toward more targeted and minimally invasive techniques, primarily involving ablative and non-ablative laser therapies.

Non-ablative approaches, such as photobiomodulation (PBM) and photodynamic therapy (PDT), have gained prominence for their ability to modulate inflammation, stimulate tissue repair, and combat pathogenic microorganisms without inducing thermal damage to surrounding tissues. These modalities leverage specific wavelengths to activate cellular processes (e.g., mitochondrial ATP production in PBM) or photosensitizers (e.g., methylene blue in PDT) to selectively target pathogens and inflammatory mediators (119).

#### 4.2.1 Photodynamic therapy

Photodynamic therapy (PDT) represents a sophisticated form of photochemotherapy, involving the precise activation of a photosensitizing agent within target tissues using light of specific wavelengths. This therapeutic modality combines three core components: a photosensitizer (a light-sensitive compound), a tailored light source (either laser or visible light), and molecular oxygen, to induce localized cytotoxic effects.

When the photosensitizer is exposed to light within its absorption spectrum, it undergoes photoexcitation, transitioning from its ground state to a triplet excited state. This excited triplet state then reacts with endogenous oxygen in the tissue, catalyzing the generation of highly reactive oxygen species (ROS), such as singlet oxygen (^1^O_2_) and hydroxyl radicals. These ROS exhibit potent cytotoxicity, causing oxidative damage to cellular membranes, proteins, and DNA, leading to cell death via apoptosis or necrosis (120, 121).

In the context of oral lichen planus (OLP), a key mechanistic advantage of PDT lies in its selective targeting of CD8 + cytotoxic T cells, which are central to the disease's immunopathogenesis. By preferentially accumulating in activated immune cells and inflamed tissues, the photosensitizer enables PDT to induce apoptosis and necrosis in these pathogenic cells, disrupting the autoimmune cascade that drives tissue damage and inflammation. This selective cytotoxicity mitigates local inflammatory processes (e.g., epithelial hyperkeratosis, subepithelial infiltrates) while preserving healthy tissue, distinguishing PDT from systemic immunosuppressive therapies (122, 123).

Clinical studies evaluating the efficacy of photodynamic therapy (PDT) in the treatment of oral lichen planus (OLP) have demonstrated consistent and significant improvements across multiple clinical parameters. These therapeutic outcomes include marked reductions in inflammation severity, pain intensity, and lesion dimensions, alongside notable enhancements in oral function and quality of life for patients.

Beyond these promising clinical results, PDT's favorable safety profile—characterized by minimal systemic toxicity, non-invasive administration, low complication rates, and negligible side effects—positions it as a viable alternative therapeutic option for OLP management. Its selective targeting of pathological tissue while preserving healthy cells underscores its potential as a precision medicine approach, particularly for patients unresponsive to conventional treatments or seeking minimally invasive interventions (46, 124–128).

Ongoing research is focused on optimizing PDT protocols, exploring synergies with adjunct therapies, and validating its long-term efficacy in diverse patient populations, further solidifying its role in the evolving landscape of OLP care (120, 129).

#### 4.2.2 Photobiomodulation

Photobiomodulation (PBM) has emerged as a promising therapeutic modality for oral lichen planus (OLP) due to its remarkable capacity to mitigate pain, resolve inflammation, and stimulate tissue regeneration.

This innovative approach harnesses the precise application of specific near-infrared (NIR) wavelengths, typically within the 600–1,000 nm range, to modulate cellular processes through photochemical interactions with endogenous chromophores such as cytochrome c oxidase (CCO), the terminal enzyme in the mitochondrial respiratory chain. When these wavelengths penetrate tissue and interact with CCO, they induce a redox reaction that disrupts the nitric oxide (NO) molecule bound to the enzyme's active site. This dissociation releases NO, reactivates CCO, and enhances its catalytic activity, leading to a significant increase in ATP production. The surplus energy generated by this process is critical for driving cellular repair mechanisms, including DNA synthesis, protein restoration, and membrane regeneration, thereby accelerating tissue healing (130).

**Figure 5:**
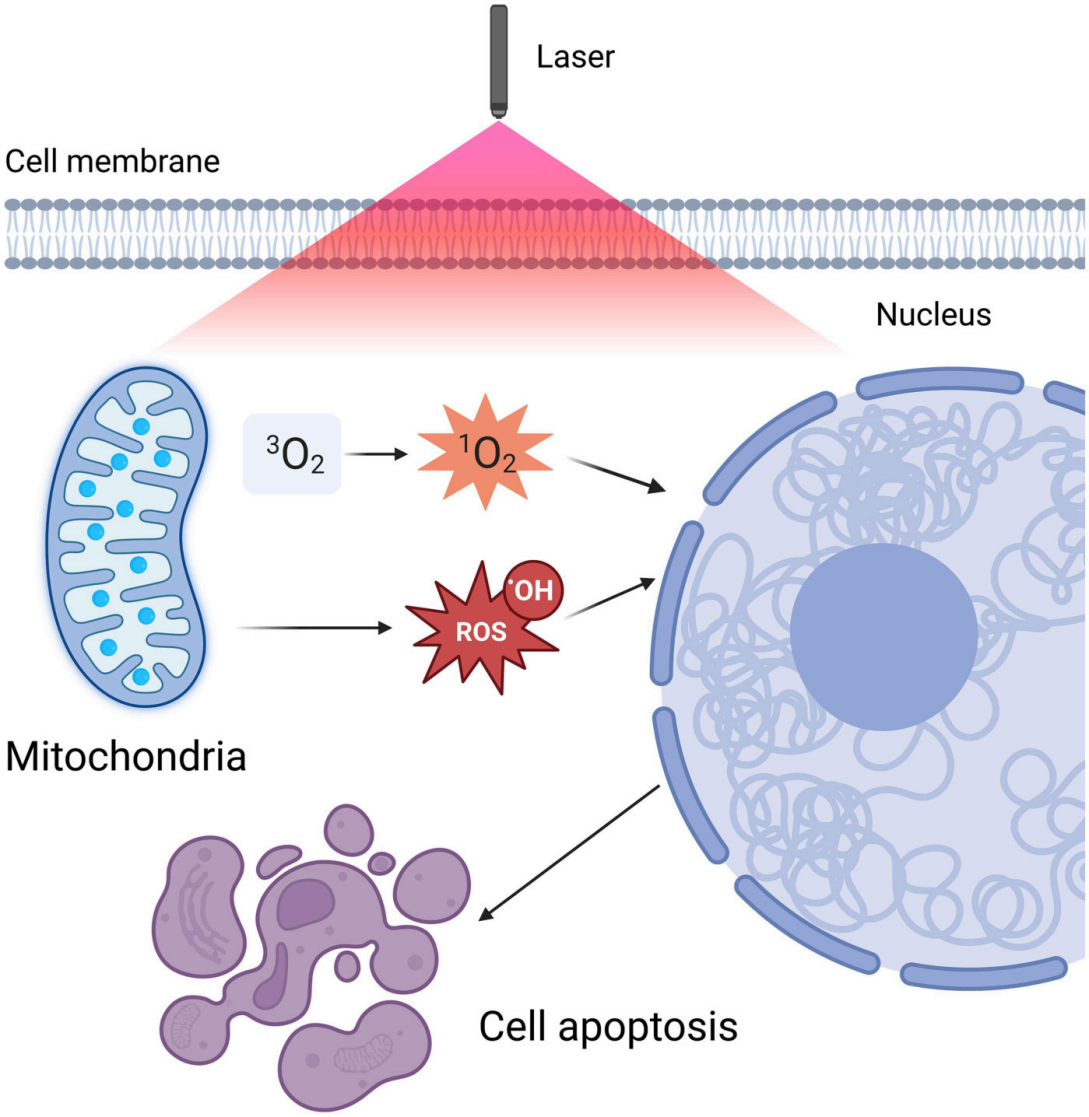
The mechanism of photobiomodulation in treating oral lichen planus.

Furthermore, PBM mitigates oxidative stress—a key driver of OLP pathogenesis—by reducing levels of reactive oxygen species (ROS). By normalizing mitochondrial function and enhancing antioxidant enzyme activity (e.g., superoxide dismutase, glutathione peroxidase), PBM neutralizes ROS, preventing cellular damage and resolving inflammation. Additionally, the therapy modulates gene expression by activating RNA transcription and DNA synthesis through upregulation of growth factors [e.g., vascular endothelial growth factor [VEGF], transforming growth factor-β [TGF-β]] and signaling pathways [e.g., mitogen-activated protein kinase (MAPK), AKT]. These molecular cascades collectively enhance cell proliferation, tissue regeneration, and immunomodulation, making PBM a versatile tool for addressing the multifaceted pathophysiology of OLP (131) (Figure 5).

Studies conducted on the effects of photobiomodulation (PBM) on oral lichen planus (OLP) have yielded promising results, shedding light on its multifaceted therapeutic mechanisms. Following PBM treatment, a notable enhancement in the expression of key cellular proteins such as bcl-2, Ki-67, and p63 within the atrophic-erosive epithelium commonly observed in OLP lesions. These proteins play crucial roles in regulating apoptosis, cell proliferation, and epithelial homeostasis, suggestting a restoration of impaired cell cycle regulation and a stimulation of keratinocyte proliferation, both of which are essential for the regeneration of damaged oral mucosa. This molecular-level recovery is believed to underpin the clinical improvements observed in patients, including epithelial thickening and accelerated wound healing, which are vital for the resolution of erosive and atrophic OLP lesions (132, 133).

In addition to promoting tissue repair, PBM has demonstrated significant analgesic properties. Research indicates that PBM can modulate peripheral nociceptive pathways by inhibiting the activity of pain receptors in the oral mucosa. This mechanism is thought to be responsible for the notable pain relief experienced by patients undergoing PBM therapy. The ability to reduce or even eliminate the need for pharmacological pain management is a particularly valuable aspect of PBM, especially in chronic conditions like OLP where persistent discomfort can significantly impact quality of life (134).

Moreover, PBM has been shown to exert anti-inflammatory and immunomodulatory effects, as evidenced by the observed reduction in erythema associated with OLP lesions. Clinical studies have demonstrated that when PBM is administered using wavelengths in the range of 630–980 nm, and power outputs between 20 and 300 mW, there is a marked decrease in the redness and inflammation of affected tissues. These parameters are believed to optimize the penetration of light into the oral mucosa, thereby enhancing mitochondrial activity and ATP synthesis, which in turn promotes tissue repair and reduces inflammatory responses (135, 136).

Importantly, numerous clinical trials and observational studies have consistently reported that PBM is a well-tolerated, safe, and non-invasive treatment modality for OLP. Unlike some conventional therapies that may involve systemic immunosuppressants or corticosteroids with potential side effects, PBM does not appear to induce any significant adverse effects when applied within recommended dosimetric parameters. This safety profile, combined with its efficacy in alleviating symptoms and promoting tissue healing, positions PBM as a promising adjunctive or standalone therapy for managing OLP (46, 64, 132, 137–140).

In conclusion, photobiomodulation offers a multi-targeted approach to treating oral lichen planus by enhancing cellular regeneration, reducing inflammation, relieving pain, and improving clinical symptoms—all without the risk of serious side effects. As research in this field continues to evolve, PBM is increasingly being recognized as a valuable tool in the comprehensive management of this challenging chronic oral condition (Table 4).

**Table 4:** Non-drug therapy in oral lichen planus.

Therapy	Reference study	Indication	Intervention	No. of pts	Duration	Frequency	Outcome measure
PRF	Bennardo (113)	OLP	1 mL PRF injections		8 wks	once a week	lesions area; VAS; Thongprasom
i-PRF	Saglam (116)	erosive OLP	i-PRF	24	60 days	four sessions; a 15-day interval between each session	lesions area; VAS; OHIP-14
i-PRF	Al-Hallak (118)	symptomatic bilateral OLP	1-mL intralesional PRF	12	4 wks	once a week	REU score; lesion areas
PRP	Hijazi (115)	erosive OLP	0.5 mL per 1 cm^2^ PRP injections	10	4 wks	once a week	Thongprasom score
PRP	Sethi (114)	erosive OLP	0.5 mL per 1 cm^2^ PRP injections	10	8 wks	once a week	VAS
PRP	ElGhareeb (117)	OLP	PRP	12	8 wks	every two weeks f	REU; NRS
PDT	Salinas-Gilabert (46)	OLP	PDT + orabase cream	20	4 wks	TID; once a week	VAS; OHIP-14; HAD; Thongprasom score
MB-PDT	Saleh (124)	erosive OLP	5%MB + focal red light (wavelength 660 nm, Intensity 100–130 m W/cm^2^)	10	4 wks	twice a week	VAS; Thongprasom score
ALA-mediated PDT	Sulewska (125)	reticular OLP	5% ALA + custom-designed diode lamp providing light at 630 nm and 300 mW	20	10 wks	once a week	VAS
PDT	Cosgarea (126)	OLP	30 s/spot, low level laser at an output power of 200 mW/cm^2^ and at an emission of 660 nm	20		day 1, 3, 7, 14	lesion size; ABSIS; Thongprasom-score
PDT + PBM	Trakarnboonki (127)	recalcitrant OLP	5% ALA + PDT + PBMT	1			
MB-PDT	Zborowski (128)	bilateral OLP	5% MB, diode laser with spot size 0.8 cm^2^ at 650 nm using energy fluence 120 J/cm^2^ and power density 1,034 mW/cm^2^ for 227 s.	15	9 days	days1, 3, 6, 9	size of lesions; Thongprasom; ABISIS; VAS
PBM	Mohamed (137)	erosive OLP	photobiomodulation therapy by 980 nm diode laser	22	5 wks	twice weekly	lesions area; VAS; MDA
PBM	Gambino (65)	erosive OLP	980/645 nm AlGaAs diode laser	10	8 wks	once a week	OCT system
PBM	Sobral (138)	OLP	diode laser 660 ± 10 nm, 100 mW, 177 J/cm^2^, 5 s, 0.5 J per point	17	30 days	three times a week	HADS; OHIP-14
PBM	Abboud (64)	symptomatic OLP	60 nm GaAIAs diode laser	17	30 days	twice a week	Pain; clinical scores; recurrence rate
PBM	Mutafchieva (132)	OLP	diode laser (810 nm), (0.50 W, 30 s, 1.2 J/cm^2^)	20	4 wks	three times a week	EI; Biopsies; analyzed immunohistochemically for p63 expression
PBM	Nammour (139)	erosive/ulcerative OLP	helium-neon red light (635 nm)	48	6 wks	every 48 h	VAS; REU; recurrence rate
PBM	Roccon (140)	OLP	A 980 nm diode laser and a flat top handpiece with a 1-cm^2^ spot area	20		once	VAS
PBM	Mutafchieva (132)	OLP	810 nm diode laser (0.5 W, 30 s, 1.2 J/cm^2^)	20	2 wks	three times a week	Biopsies; VAS; Thongprasom; analyzed immunohistochemically for bcl-2 and Ki-67 expression

### 4.3 Psychosocial interventions

The European S1 guidelines pionted out that stress and anxiety are potential risk factors for the pathogenesis of oral lichen planus (OLP), although the precise nature of this correlation remains a subject of debate (141).

Specific psychological traits and emotional regulation patterns play a pivotal role in the pathogenesis and exacerbation of OLP (142). Psychological assessments utilizing the Hamilton Anxiety Scale and the Montgomery-Asberg Depression Rating Scale were administered to evaluate the cohort diagnosed with OLP. The results indicated that both anxiety and depression serve as significant risk factors that may modulate the progression of OLP (143). Depressive symptoms and low self-control are particularly associated with the reticular-papular subtype (144). There was a significant positive correlation between the trait of Reactional Anger and the severity of OLP-associated pain. Furthermore, the suppression of anger and its internal control characterized by the accumulation of tension may trigger or aggravate the condition (145).

While investigation into salivary alpha-amylase as a stress biomarker revealed no significant difference between OLP patients and controls, a marked disparity was evident in psychological well-being (146). Compared with healthy individuals, OLP patients are at an increased risk for anxiety, and this risk is further amplified in female patients (147). OLP patients, regardless of the clinical subtype, demonstrate significantly higher Perceived Stress Scale scores, which influence development or increase of the anxiety and depression and may decrease patients' quality of life (148).

This psychopathological state not only precipitates emotional distress but also leads to a marked deterioration in Quality of Life, impairing physical health, vitality, mental well-being, and social functioning. Notably, a vicious cycle often emerges wherein prolonged disease duration and persistent symptoms exacerbate perceived stress, thereby heightening anxiety, depression, and the tendency toward catastrophizing.

Given the profound impact of psychological factors on prognosis and recurrence, a multidimensional, comprehensive intervention strategy is imperative. Incorporating routine psychological screening, stress management, and necessary psychiatric collaboration into the standard management protocol for OLP is essential to halt disease progression and enhance overall therapeutic efficacy by addressing the patient's mental well-being.

## 5 Summary and outlook

**Figure 6:**
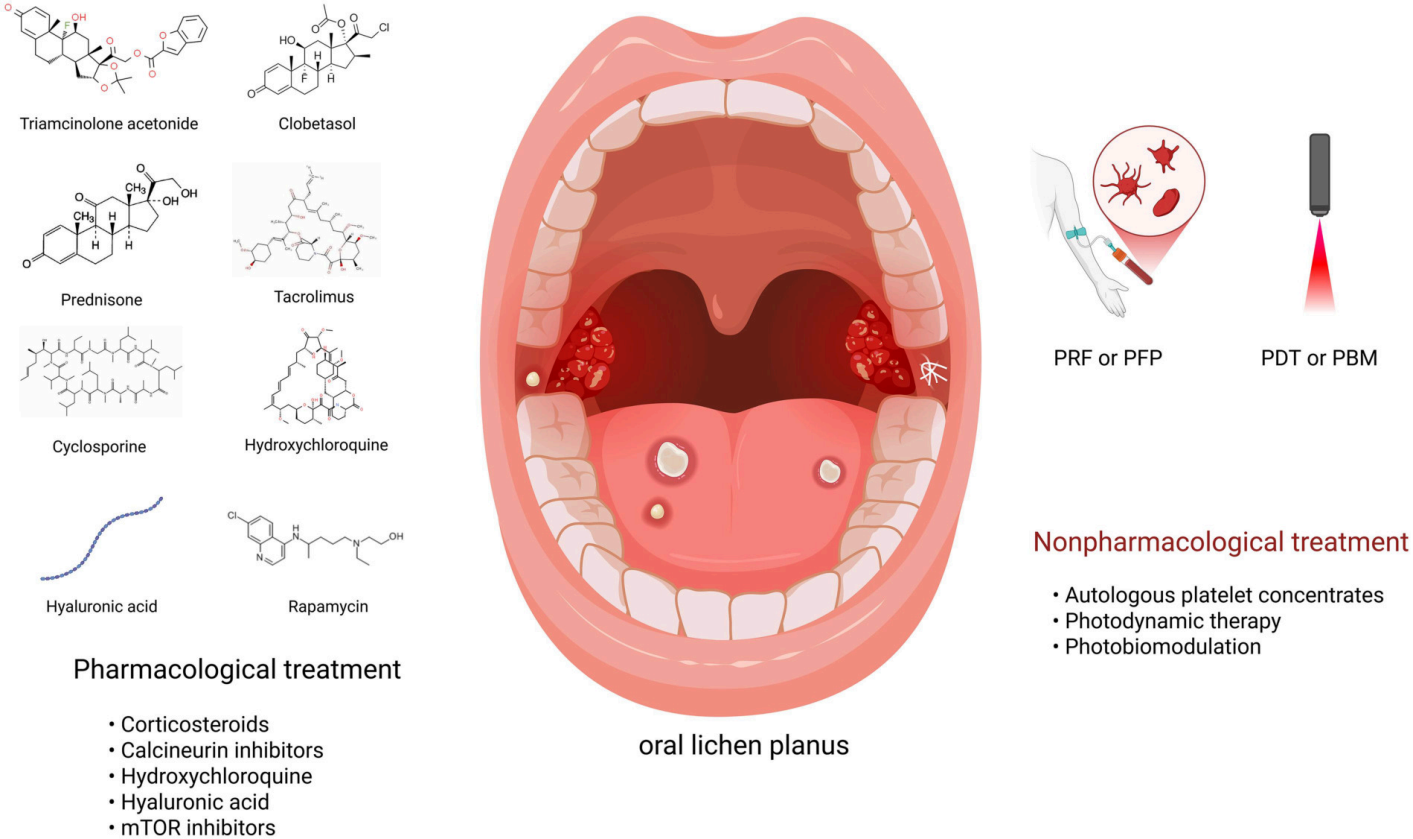
Pharmacological treatments and nonpharmacological treatments of oral lichen planus.

In 2020, the European S1 guidelines issued treatment recommendations for OLP (141). This paper reviews 46 pieces of literature published after 2020 regarding OLP drug and non-drug therapies, and summarizes the conventional treatment medications for OLP, including: corticosteroids, calcineurin inhibitors, hydroxychloroquine, hyaluronic acid, mTOR inhibitors, and non-pharmacological treatment approaches such as autologous platelet concentrates, photodynamic therapy, and photobiomodulation (Figure 6).

Corticosteroids represent the primary pharmacological intervention for the management of OLP, encompassing a diverse array of agents including triamcinolone acetonide and clobetasol. The application of these steroids, both topically and through submucosal injection, is considered suitable for the treatment of mild to moderate lesions, whereas systemic administration, often involving prednisone, is typically employed for refractory and severe multifocal presentations. However, it is important to note that the use of corticosteroids may be associated with adverse effects, such as fungal infections. Calcinurin inhibitors, such as tacrolimus and cyclosporine, are frequently employed in the treatment of OLP patients who exhibit resistance to corticosteroid therapy, particularly in cases of refractory erosive oral lichen planus. Nevertheless, the utilization of tacrolimus may give rise to transient burning sensations or painful side effects, and the excessive use of cyclosporine can potentially lead to gingival hyperplasia. Hydroxychloroquine can alleviate symptoms of OLP, but prolonged use may result in retinal damage. Short-term local application of hyaluronic acid is effective in managing OLP, yet its capacity to contribute to long-term disease control remains a topic of contention. The mTOR inhibitor rapamycin has a therapeutic effect on EOLP, albeit it is associated with adverse reactions such as bone marrow suppression and hyperlipidemia.

Based on the above literature review, proposals for optimizing and replacing existing drug treatments are as following:

**Combination Therapy:** plementing combination therapy strategies that involve currently used drugs for OLP in conjunction with different types of medications has shown greater efficacy compared to monotherapy. This approach holds significant potential in enhancing treatment outcomes for patients with OLP.

**Autologous Blood Products:** Therapies utilizing autologous blood products such as Platelet-Rich Plasma (PRP) and Platelet-Rich Fibrin (PRF) exhibit promising results as alternative treatment options for OLP. These therapies offer a more sustainable and safer approach, with reduced reliance on conventional treatments and their associated side effects.

**Non-Pharmacological Treatments:** Non-pharmacological interventions such as Photodynamic Therapy (PDT) and Photobiomodulation (PBM) demonstrate low toxicity, invasiveness, and complications, along with minimal side effects. These modalities should indeed be considered as viable alternative therapies for OLP, offering a less invasive and potentially more effective treatment avenue for patients. Concomitant psychological screening and intervention can significantly improve therapeutic outcomes in patients with OLP.

In conclusion, this article has conducted an extensive review of the literature, summarizing the characteristics and recent advancements in drug therapy and drug replacement therapy for OLP. By analyzing current treatment modalities, we have proposed innovative suggestions for enhancing existing therapy. These insights offer new perspectives on drug combination strategies and potential drug replacements for OLP treatment. Future research and clinical trials should focus on validating and optimizing these approaches, to develop more effective and personalized treatment plans for individuals affected by OLP.

## References

[1] Nukaly HY Halawani IR Alghamdi SM Alruwaili AG Binhezaim A Algahamdi RA. Oral lichen Planus: a narrative review navigating etiologies, clinical manifestations, diagnostics, and therapeutic approaches. J Clin Med. (2024) 13:5280. 10.3390/jcm13175280 39274493 PMC11396719

[2] Li C Tang X Zheng X Ge S Wen H Lin X. Global prevalence and incidence estimates of oral lichen Planus: a systematic review and meta-analysis. JAMA Dermatol. (2020) 156:172–81. 10.1001/jamadermatol.2019.3797 31895418 PMC6990670

[3] Sandhu S Klein BA Al-Hadlaq M Chirravur P Bajonaid A Xu Y. Oral lichen planus: comparative efficacy and treatment costs-a systematic review. BMC Oral Health. (2022) 22:161. 10.1186/s12903-022-02168-4 35524296 PMC9074269

[4] Wang H Zhang D Han Q Zhao X Zeng X Xu Y. Role of distinct CD4(+) T helper subset in pathogenesis of oral lichen planus. Oral Pathol Med. (2016) 45:385–93. 10.1111/jop.12405 26693958

[5] Kurago ZB. Etiology and pathogenesis of oral lichen planus: an overview. Oral Surg Oral Med Oral Pathol Oral Radiol. (2016) 122:72–80. 10.1016/j.oooo.2016.03.011 27260276

[6] Giuliani M Troiano G Cordaro M Corsalini M Gioco G Muzio LL. Rate of malignant transformation of oral lichen planus: a systematic review. Oral Dis. (2019) 25:693–709. 10.1111/odi.12885 29738106

[7] Wang D Shaiba S Woo S-B. A guide for dental practitioners of common oral potentially malignant disorders. J Calif Dent Assoc. (2021) 49:223–36. 10.1080/19424396.2021.12222696

[8] Novembre D Barca I Cordaro R Kallaverja E Ferragina F Cristofaro MG. Malignant transformation of oral lichen planus. A retrospective analysis from 2003 to 2014: our experience. Ann Ital Chir. (2020) 91:445–50. 33295299

[9] Oberti L Gabrione F Lucchese A Stasio DD Lauritano D. Treatment of oral lichen planus: a narrative review. Front Physiol. (2019) 10. 10.3389/conf.fphys.2019.27.00004

[10] Torrente-Castells E Figueiredo R Berini-Aytés L Gay-Escoda C. Clinical features of oral lichen planus. A retrospective study of 65 cases. Med Oral Patol Oral Cir Bucal. (2010) 15:e685–90. 10.4317/medoral.15.e685 20383118

[11] Shah B Ashok L Sujatha GP. Evaluation of salivary cortisol and psychological factors in patients with oral lichen planus. Indian J Dent Res. (2009) 20:288–92. 10.4103/0970-9290.57361 19884710

[12] Girardi C Luz C Cherubini K Figueiredo MAZD Nunes MLT Salum FGA. Salivary cortisol and dehydroepiandrosterone (DHEA) levels, psychological factors in patients with oral lichen planus. Arch Oral Biol. (2011) 56:864–8. 10.1016/j.archoralbio.2011.02.003 21377142

[13] Ghapanchi J Ghaderi H Haghshenas MR Jamshidi S Rezazadehf F Azad A. Observational molecular case-control study of genetic polymorphisms 1 in programmed cell death protein-1 in patients with oral lichen Planus. Asian Pac J Cancer Prev. (2019) 20:421–4. 10.31557/APJCP.2019.20.2.421 30803202 PMC6897023

[14] Albu C-C Bencze A Dragomirescu A-O Vlădan C Albu Ş-D Russu E-A. Oral lichen planus genetics update. Eur J Dent Oral Health. (2022) 3:1–5. 10.24018/ejdent.2022.3.4.205

[15] Elenbaas A Enciso R Al-Eryani K. Oral lichen planus: a review of clinical features, etiologies, and treatments. Dent Rev. (2022) 2:100007. 10.1016/j.dentre.2021.100007

[16] Lu SL Qi XM Dong G Chen SL Guo DW Wang YL. Clinical characteristics and analysis of familial oral lichen planus in eight Chinese families. Exp Ther Med. (2016) 12:2281–4. 10.3892/etm.2016.3597 27698724 PMC5038863

[17] Shen H Liu Q Huang P Fan H Zang F Liu M. Vitamin D receptor genetic polymorphisms are associated with oral lichen planus susceptibility in a Chinese Han population. BMC Oral Health. (2020) 20:26. 10.1186/s12903-020-1002-3 32000758 PMC6993400

[18] Yu FY Wang QQ Li M Cheng YH Cheng YL Zhou Y. Dysbiosis of saliva microbiome in patients with oral lichen planus. BMC Microbiol. (2020) 20:75. 10.1186/s12866-020-01733-7 32245419 PMC7118920

[19] Chen J Liu K Sun X Shi X Zhao G Yang Z. Microbiome landscape of lesions and adjacent normal mucosal areas in oral lichen planus patient. Front Microbiol. (2022) 13:992065. 10.3389/fmicb.2022.992065 36338092 PMC9630593

[20] Ciesielska AA-O Kusiak AA-O Ossowska A Grzybowska MA-O. Changes in the oral cavity in menopausal women-A narrative review. Int J Environ Res Public Health. (2021) 19:253. 10.3390/ijerph19010253 35010513 PMC8750983

[21] Khudhur AS Di Zenzo G Carrozzo M. Oral lichenoid tissue reactions: diagnosis and classification. Expert Rev Mol Diagn. (2014) 14:169–84. 10.1586/14737159.2014.888953 24524807

[22] Cinar G Metin A. Carbamazepine-Induced oral lichenoid reaction: a report of a rare case. Cureus. (2025) 17:e81835. 10.7759/cureus.81835 40337574 PMC12057648

[23] Payeras MR Cherubini K Figueiredo MA Salum FG. Oral lichen planus: focus on etiopathogenesis. Arch Oral Biol. (2013) 58:1057–69. 10.1016/j.archoralbio.2013.04.004 23660124

[24] Lavanya N Jayanthi P Rao UK Ranganathan K. Oral lichen planus: an update on pathogenesis and treatment. J Oral Maxillofac Pathol. (2011) 15:127–32. 10.4103/0973-029X.84474 22529568 PMC3329692

[25] Teja R Devy S Nirmal M Pm S. Oral lichen Planus: a review of etiopathogenesis, clinical, histological and treatment aspects. J Interdiscipl Med Dent Sci. (2014) 2:2–5. 10.4172/2376-032X.1000142

[26] Ibrahim ZAE El-Ashmawy AA Neinaa YME Mohammad DAE. Immunohistochemical expression of calmodulin in cutaneous lichen Planus: a case-control study. Indian J Dermatol. (2019) 64:338. 10.4103/ijd.IJD_91_18 31516153 PMC6714185

[27] Mehrbani SP Motahari P Azar FP Ahari MA. Role of interleukin-4 in pathogenesis of oral lichen planus: a systematic review. Med Oral Patol Oral Cir Bucal. (2020) 25:e410–5. 10.4317/medoral.23460 32134902 PMC7211366

[28] Parlatescu I Tovaru M Nicolae CL Sfeatcu RA-O Didilescu AC. Oral health-related quality of life in different clinical forms of oral lichen planus. Clin Oral Investig. (2020) 24:301–8. 10.1007/s00784-019-02951-8 31098713

[29] Cox TA-O Woodhead J Nelson BL. Reticular oral lichen Planus. Head Neck Pathol. (2020) 14:192–4. 10.1007/s12105-018-0983-6 30390195 PMC7021865

[30] Patigaroo SA Ali I Maqbool T Qadri H Showkat SA Latoo MA. Reticular oral lichen Planus: a clinical experience of ENT surgeons. Indian J Otolaryngol Head Neck Surg. (2023) 75:390–6. 10.1007/s12070-022-03267-y 37275004 PMC10235395

[31] Dalirsani Z Seyyedi SA. Treatment of plaque-like oral lichen Planus with CO2 Laser. Indian J Dermatol. (2021) 66:698–703. 10.4103/ijd.ijd_1170_20 35283528 PMC8906308

[32] Leteurtre NH Sebastián JB Tortajada FC De Miguel EL Soriano YJ Alegría JB. Oral lichen planus plaques and homogeneous leukoplasia: comparative results of treatment with CO2 laser. Acta Otorrinolaringol Esp. (1999) 50:543–7. 10619881

[33] Gupta S Jawanda MK. Oral lichen Planus: an update on etiology, pathogenesis, clinical presentation, diagnosis and management. Indian J Dermatol. (2015) 60:222–9. 10.4103/0019-5154.156315 26120146 PMC4458931

[34] Palaniappan P Baalann KP. Erosive oral lichen planus. Pan Afr Med J. (2021) 40:73. 10.11604/pamj.2021.40.73.26013 34804341 PMC8590277

[35] Leroy C Lesclous P Dutot N Anquetil M Tessier MH. Erosive oral lichen planus: think thymoma. Ann Dermatol Venereol. (2023) 150:152–4. 10.1016/j.annder.2022.11.004 36653225

[36] Rogers RS III Eisen D. Erosive oral lichen planus with genital lesions: the vulvovaginal-gingival syndrome and the peno-gingival syndrome. Dermatol Clin. (2003) 21:91–8. 10.1016/S0733-8635(02)00059-1 12622271

[37] Weston G Payette M. Update on lichen planus and its clinical variants. Int J Womens Dermatol. (2015) 1:140–9. 10.1016/j.ijwd.2015.04.001 28491978 PMC5418875

[38] Boorghani M Gholizadeh N Zenouz AT Vatankhah M Mehdipour M. Oral lichen planus: clinical features, etiology, treatment and management; a review of literature. J Dent Res Dent Clin Dent Prospects. (2010) 4:3–9. 10.5681/joddd.2010.002 22991586 PMC3429956

[39] Chiang CP Yu-Fong Chang J Wang YP Wu YH Lu SY Sun A. Oral lichen planus—differential diagnoses, serum autoantibodies, hematinic deficiencies, and management. J Formos Med Assoc. (2018) 117:756–65. 10.1016/j.jfma.2018.01.021 29472048

[40] Gobbo M Rupel K Zoi V Perinetti G Ottaviani G Di Lenarda R. Scoring systems for oral lichen Planus used by differently experienced raters. Med Oral Patol Oral Cir Bucal. (2017) 22:e562–71. 10.4317/medoral.21833 28809373 PMC5694178

[41] Slade GD. Derivation and validation of a short-form oral health impact profile. Community Dent Oral Epidemiol. (1997) 25:284–90. 10.1111/j.1600-0528.1997.tb00941.x 9332805

[42] Saberi Z Tabesh A Darvish S. Oral health-related quality of life in erosive/ulcerative oral lichen planus patients. Dent Res J (Isfahan). (2022) 19:55. 10.4103/1735-3327.351344 36159060 PMC9490254

[43] Park HK Hurwitz S Woo SB. Oral lichen planus: REU scoring system correlates with pain. Oral Surg Oral Med Oral Pathol Oral Radiol. (2012) 144:75–82. 10.1016/j.oooo.2012.02.013 22727095

[44] Ormond MA-O McParland H Thakrar P Donaldson ANA Andiappan M Cook RJ. Validation of an oral disease severity score (ODSS) tool for use in oral mucous membrane pemphigoid. Br J Dermatol. (2020) 183:78–85. 10.1111/bjd.18566 31571192

[45] Selaru CA Parlatescu I Milanesi E Dobre M Tovaru S. Impact of altered lipid profile in oral lichen Planus. Maedica (Bucur). (2023) 18:12–8. 10.26574/maedica.2023.18.1.19 37266475 PMC10231171

[46] Salinas-Gilabert C Gómez García FA-O Galera Molero F Pons-Fuster EA-O Vander Beken SA-O Lopez Jornet PA-O. Photodynamic therapy, photobiomodulation and acetonide triamcinolone 0.1% in the treatment of oral lichen Planus: a randomized clinical trial. Pharmaceutics. (2022) 15:30. 10.3390/pharmaceutics15010030 36678659 PMC9862179

[47] Bender A Fix C Eubel V Eming R Pollmann R Schmidt T. Adjuvant high-dose intravenous immunoglobulins for recalcitrant erosive oral lichen planus: mixed clinical responses. Eur J Dermatol. (2018) 28:496–501. 10.1684/ejd.2018.3370 30325320

[48] Ayyar VS Jusko WJ. Transitioning from basic toward systems pharmacodynamic models: lessons from corticosteroids. Pharmacol Rev. (2020) 72:414–38. 10.1124/pr.119.018101 32123034 PMC7058984

[49] Lavaee FA-O Shadmanpour M. Comparison of the effect of photodynamic therapy and topical corticosteroid on oral lichen planus lesions. Oral Dis. (2019) 25:1954–63. 10.1111/odi.13188 31478283

[50] Ofluoglu D Ergun S Warnakulasuriya S Namdar-Pekiner F Tanyeri H. An evaluation of the efficacy of a topical gel with triester glycerol oxide (TGO) in the treatment of minor recurrent aphthous stomatitis in a Turkish cohort: a randomized, double-blind, placebo-controlled clinical trial. Med Oral Patol Oral Cir Bucal. (2017) 22:e159–66. 10.4317/medoral.21469 28160585 PMC5359701

[51] ElGhareeb MI ElMokadem S Elsherif M ElKashishy K. Interleukin 9 in oral lichen planus: an immunohistochemical study before and after treatment by intralesional steroid injection. Arch Dermatol Res. (2024) 316:742. 10.1007/s00403-024-03452-9 39499320

[52] Riyaz SMA. Efficacy of clobetasol propionate (0.05%), amlexanox (5%), and triamcinolone acetonide (0.1%) in the treatment of oral lichen planus. J Pharm Bioallied Sci. (2024) 16:S736–7. 10.4103/jpbs.jpbs_984_23 38595401 PMC11000870

[53] Bajoria AA Chinnannavar SN Mishra S Singh DK Pathi J Jha VK. Comparative evaluation of pimecrolimus cream 1% and triamcinolone aceonide paste in treatment of atrophic-erosive oral lichen planus. J Pharm Bioallied Sci. (2023) 15:S1274–6. 10.4103/jpbs.jpbs_127_23 37694075 PMC10485432

[54] Kaur M Gulati P Verma S Khare P Garg R Dutta J. Efficacy of Aloe Vera, amlexanox (5%), and triamcinolone acetonide (0.1%) in the management of oral lichen Planus. J Pharm Bioallied Sci. (2024) 16:S133–5. 10.4103/jpbs.jpbs_416_23 38595509 PMC11000937

[55] Vinay K Kumar S Dev A Cazzaniga S Borradori L Thakur V. Oral Acitretin plus topical triamcinolone vs topical triamcinolone monotherapy in patients with symptomatic oral lichen planus: a randomized clinical trial. JAMA Dermatol. (2024) 160:80–7. 10.1001/jamadermatol.2023.4889 38055232 PMC10701665

[56] Lee YC Lee JS Jung AR Park JM Eun YG. Factors affecting the result of intralesional corticosteroid injection in patients with oral lichen planus. Clin Exp Otorhinolaryngol. (2018) 11:205–9. 10.21053/ceo.2017.01319 29366304 PMC6102332

[57] Zhao W Lin D Deng SA-O Wang S Guo Y Yang J. Synergistic efficacy of plaque control with intralesional triamcinolone acetonide injection on erosive non-gingival oral lichen planus: a randomized controlled clinical trial. Int J Environ Res Public Health. (2022) 19:13787. 10.3390/ijerph192113787 36360666 PMC9655481

[58] Zeng Q Peng S Bai X Chen J Liu B Pan H. A case of delayed allergic reaction caused by local injection of triamcinolone acetonide. Zhong nan da xue xue bao. Yi xue ban=J Cent South Univ Med Sci. (2020) 45:216–20. 10.11817/j.issn.1672-7347.2020.180584 32386051

[59] Sneha DS Nisa DSU Mhapuskar A. Curcumin—a novel ayurvedic treatment for oral lichen planus. IJCPR Int J Current Pharm Rev Res. (2017) 3:1507–11. 10.24327/23956429.ijcmpr20170021

[60] Bakhshi MA-O Gholami S Mahboubi A Jaafari MR Namdari M. Combination therapy with 1% nanocurcumin gel and 0.1% triamcinolone acetonide mouth rinse for oral lichen planus: a randomized double-blind placebo controlled clinical trial. Dermatol Res Pract. (2020) 2020:4298193. 10.1155/2020/4298193 32518558 PMC7256737

[61] Agha-Hosseini F Atyabi F Akbari K Moosavi MS. Decreased recurrence of symptoms in oral lichen planus with intralesional injection of hyaluronic acid and triamcinolone. Int J Oral Maxillofac Surg. (2021) 50:1643–8. 10.1016/j.ijom.2021.02.028 33741218

[62] Rodriguez-Galvez R Tvarijonaviciute A Peres-Rubio C Lopez-Jornet PA-O. Efficacy of combined vs. monotherapy in oral lichen planus: a randomized clinical trial. Oral Dis. (2025) 31:522–31. 10.1111/odi.15151 39435599 PMC11976123

[63] Chamani G Rad M Zarei MR Lotfi S Sadeghi M Ahmadi Z. Efficacy of tacrolimus and clobetasol in the treatment of oral lichen planus: a systematic review and meta-analysis. Int J Dermatol. (2015) 54:996–1004. 10.1111/ijd.12925 26204904

[64] Abboud CS Brandão E Cunha KRL de Sousa Brito K Gallo CB Molon AA-O. Serum and salivary cytokines in patients with oral lichen planus treated with photobiomodulation. Oral Dis. (2023) 29:1250–8. 10.1111/odi.14084 34817126

[65] Gambino A Cabras M Cafaro A Broccoletti R Carossa S Hopper C. Preliminary evaluation of the utility of optical coherence tomography in detecting structural changes during photobiomodulation treatment in patients with atrophic-erosive oral lichen planus. Photodiagnosis Photodyn Ther. (2021) 34:102255. 10.1016/j.pdpdt.2021.102255 33727132

[66] Carbone M Goss E Carrozzo M Castellano S Conrotto D Broccoletti R. Systemic and topical corticosteroid treatment of oral lichen planus: a comparative study with long-term follow-up. J Oral Pathol Med. (2003) 32:323–9. 10.1034/j.1600-0714.2003.00173.x 12787038

[67] Muzio LL Della Valle A Mignogna MD Pannone G Bucci P Bucci E. The treatment of oral aphthous ulceration or erosive lichen planus with topical clobetasol propionate in three preparations: a clinical and pilot study on 54 patients. J Oral Pathol Med. (2001) 30:611–7. 10.1034/j.1600-0714.2001.301006.x 11722711

[68] Vaidya AU Khorate MM Chinam N Figueiredo N. Efficacy of Aloe Vera and clobetasol propionate in the management of oral lichen planus: a randomized parallel clinical trial. Front Dent. (2023) 20:4. 10.18502/fid.v20i4.12358 37312826 PMC10258394

[69] Kamal YA-O Abdelwhab AA-O Salem SA-O Fakhr M. Evaluation of the efficacy of supplementary probiotic capsules with topical clobetasol propionate 0.05% versus topical clobetasol propionate 0.05% in the treatment of oral lichen planus (a randomized clinical trial). BMC Oral Health. (2025) 25:344. 10.1186/s12903-024-05246-x 40045332 PMC11884111

[70] Santonocito S Polizzi AA-O De Pasquale R Ronsivalle VA-O Lo Giudice AA-O Isola GA-O. Analysis of the efficacy of two treatment protocols for patients with symptomatic oral lichen planus: a randomized clinical trial. Int J Environ Res Public Health. (2020) 18:56. 10.3390/ijerph18010056 33374791 PMC7794703

[71] Schroeder FMM Pedraça ES Palma VM Carrard VC Martins MAT Maito F. Topical tacrolimus orabase versus topical clobetasol propionate orabase in the treatment of symptomatic oral lichen planus: a pilot randomized study. Clin Oral Investig. (2024) 28:559. 10.1007/s00784-024-05943-5 39348002

[72] Mamadapur R Naik Z Kumar SL Bagewadi A. Comparative efficacy of topical coconut cream and clobetasol propionate ointment for the management of oral lichen planus: a double-blinded randomized control trial. Indian J Pharmacol. (2022) 54:84–9. 10.4103/ijp.ijp_984_20 35546458 PMC9249154

[73] George S Balan A. A potential side effect of oral topical steroids: central serous chorioretinopathy. Indian J Dent Res. (2018) 29:107–8. 10.4103/ijdr.IJDR_694_16 29442094

[74] Einarsdottir MA-O Bankvall MA-O Robledo-Sierra JA-O Rödström PA-O Bergthorsdottir RA-O Trimpou PA-OX. Topical clobetasol treatment for oral lichen planus can cause adrenal insufficiency. Oral Dis. (2024) 30:1304–12. 10.1111/odi.14588 37103329

[75] Lodi G Tarozzi M Sardella A Demarosi F Canegallo L Di-Benedetto D. Miconazole as adjuvant therapy for oral lichen planus: a double-blind randomized controlled trial. Br J Dermatol. (2007) 156:1336–41. 10.1111/j.1365-2133.2007.07883.x 17535232

[76] Wang H Luo J Luo K Wu L Hu T Yang J. Glycyrrhizin alleviates the toxicity of hydroxychloroquine in treating oral lichen planus by occupying heat shock protein 90 alpha. Phytomedicine. (2024) 135:156059. 10.1016/j.phymed.2024.156059 39550918

[77] Jiang L Huang Y Fang M Chen X Feng D Liu J. Dynamic changes of Th1/Th2/Th17 cytokines and hBD-2/3 in erosive oral lichen planus patients saliva before and after prednisone acetate treatment. Heliyon. (2024) 10:e24043. 10.1016/j.heliyon.2024.e24043 38283247 PMC10818186

[78] AlMutairi M Riyaz SMA Awinashe M Almutairi FJ. Assessment of 5% amlexanox, 0.1% triamcinolone acetonide and 0.03% tacrolimus in the management of oral lichen planus. J Pharm Bioallied Sci. (2023) 15:S1298–300. 10.4103/jpbs.jpbs_112_23 37693973 PMC10485504

[79] Walia C Rallan NS Premkumar A Roy S. Clinical evaluation of efficacy of triamcinolone acetonide with tacrolimus in the management of oral lichen planus: a pilot prospective observational study. Contemp Clin Dent. (2022) 13:236–41. 10.4103/ccd.ccd_899_20 36213860 PMC9533377

[80] Wu T Bai Y Jing Y Chen F. What can we learn from treatments of oral lichen planus? Front Cell Infect Microbiol. (2024) 14:1279220. 10.3389/fcimb.2024.1279220 38426013 PMC10902003

[81] Yang H Wu Y Ma H Jiang L Zeng X Dan H. Possible alternative therapies for oral lichen planus cases refractory to steroid therapies. Oral Surg Oral Med Oral Pathol Oral Radiol. (2016) 121:496–509. 10.1016/j.oooo.2016.02.002 27068310

[82] Zhang Y Lin M Zhang S Wang Z Jiang L Shen J. NF-kappaB-dependent cytokines in saliva and serum from patients with oral lichen planus: a study in an ethnic Chinese population. Cytokine. (2008) 41:144–9. 10.1016/j.cyto.2007.11.004 18222093

[83] Tao XA Xia J Chen XB Wang H Dai YH Rhodus NL. FOXP3T Regulatory cells in lesions of oral lichen planus correlated with disease activity. Oral Dis. (2010) 16:76–82. 10.1111/j.1601-0825.2009.01608.x 19650850

[84] Su Z Lu J Ling Z Li W Yang X Cheng B. Upregulation of IL-37 in epithelial cells: a potential new mechanism of T cell inhibition induced by tacrolimus. Biochem Pharmacol. (2023) 216:115796. 10.1016/j.bcp.2023.115796 37690572

[85] Tobon-Arroyave SI Villegas-Acosta FA Ruiz-Restrepo SM Vieco-Duran B Restrepo-Misas M Londoño-López ML. Expression of caspase-3 and structural changes associated with apoptotic cell death of keratinocytes in oral lichen planus. Oral Dis. (2004) 10:173–8. 10.1046/j.1601-0825.2003.00998.x 15089928

[86] Ibrahim SS Ragy NI Nagy NA-O El-Kammar H Elbakry AM Ezzatt OA-O. Evaluation of muco-adhesive tacrolimus patch on caspase-3 induced apoptosis in oral lichen planus: a randomized clinical trial. BMC Oral Health. (2023) 23:99. 10.1186/s12903-023-02803-8 36788511 PMC9930326

[87] Bagan J Compilato D Paderni C Campisi G Panzarella V Picciotti M. Topical therapies for oral lichen planus management and their efficacy: a narrative review. Curr Pharm Des. (2012) 18:5470–80. 10.2174/138161212803307617 22632394

[88] Kiyani A Sohail KA-O Saeed MHB. Efficacy of 0.1% tacrolimus in long-term management of erosive lichen planus. J Dermatolog Treat. (2021) 32:367–71. 10.1080/09546634.2019.1654072 31390902

[89] Huang C Li F. Effectiveness of platelet-rich plasma gel alongside tacrolimus ointment in managing erosive oral lichen planus and its effect on oral immune microenvironment. Pak J Pharm Sci. (2024) 37:1625–33. 39923155

[90] Arguelles AR Gorbea RM Zamora MI Crelgo JG. Topic tacrolimus, alternative treatment for oral erosive lichen planus resistant to steroids: a case report. Med Oral Patol Oral Cir Bucal. (2006) 11:E462–466. 17072247

[91] Monshi BA-O Ellersdorfer C Edelmayer M Dvorak G Ganger C Ulm C. Topical cyclosporine in oral lichen planus-A series of 21 open-label, biphasic, single-patient observations. J Clin Med. (2021) 10:5454. 10.3390/jcm10225454 34830736 PMC8622196

[92] Georgaki M Piperi E Theofilou VI Pettas E Stoufi E Nikitakis NG. A randomized clinical trial of topical dexamethasone vs. Cyclosporine treatment for oral lichen planus. Med Oral Patol Oral Cir Bucal. (2022) 27:e113–24. 10.4317/medoral.25040 34564686 PMC8898582

[93] Sun SA-O Liu JJ Zhong B Wang JK Jin X Xu H. Topical calcineurin inhibitors in the treatment of oral lichen planus: a systematic review and meta-analysis. Br J Dermatol. (2019) 181:1166–76. 10.1111/bjd.17898 30903622

[94] Ribero S Stieger M Quaglino P Hongang T Bornstein MM Naldi L. Efficacy of topical tacrolimus for oral lichen planus: real-life experience in a retrospective cohort of patients with a review of the literature. J Eur Acad Dermatol Venereol. (2015) 29:1107–13. 10.1111/jdv.12758 25308924

[95] Zisis V Andreadis D Karpouzi R Karadagli T Poulopoulos A. Cyclosporine-induced gingival hyperplasia in a patient with lichen planopilaris: misfortunes never come singly!. Cureus. (2023) 15:e42531. 10.7759/cureus.42531 37637542 PMC10458405

[96] Nanda T Singh B Sharma P Arora KA-O. Cyclosporine A and amlodipine induced gingival overgrowth in a kidney transplant recipient: case presentation with literature review. BMJ Case Rep. (2019) 12:e229587. 10.1136/bcr-2019-229587 31142490 PMC6557534

[97] Lundström IM Anneroth GB Holmberg K. Candida in patients with oral lichen planus. Int J Oral Surg. (1984) 13:226–38. 10.1016/S0300-9785(84)80008-3 6430830

[98] Eisen D. Hydroxychloroquine sulfate (plaquenil) improves oral lichen planus: an open trial. J Am Acad Dermatol. (1993) 28:609–12. 10.1016/0190-9622(93)70082-5 8463463

[99] Chirravur P Sroussi H Treister N Al Hadlaq M Whiting B Santoianni JA. Hydroxychloroquine for the management of recalcitrant oral lichen planus. Oral Surg Oral Med Oral Pathol Oral Radiol. (2024) 137:355–61. 10.1016/j.oooo.2023.12.007 38278674

[100] Schrezenmeier E Dörner TA-O. Mechanisms of action of hydroxychloroquine and chloroquine: implications for rheumatology. Nat Rev Rheumatol. (2020) 16:155–66. 10.1038/s41584-020-0372-x 32034323

[101] Raj SC Baral D Garhnayak L Mahapatra A Patnaik K Tabassum S. Hydroxychloroquine-A new treatment option for erosive oral lichen planus. Indian J Dent Res. (2021) 32:192–8. 10.4103/ijdr.IJDR_943_20 34810388

[102] Shetty RR Burde KN Guttal KS. The efficacy of topical hyaluronic acid 0.2% in the management of symptomatic oral lichen planus. J Clin Diagn Res. (2016) 10:ZC46–50. 10.7860/JCDR/2016/15934.7101 26894175 PMC4740703

[103] Hashem AS Issrani RA-O Elsayed TEE Prabhu N. Topical hyaluronic acid in the management of oral lichen planus: a comparative study. J Investig Clin Den. (2019) 10:e12385. 10.1111/jicd.12385 30556961

[104] Fogel AL Hill S Teng JM. Advances in the therapeutic use of mammalian target of rapamycin (mTOR) inhibitors in dermatology. J Am Acad Dermatol. (2015) 72:879–89. 10.1016/j.jaad.2015.01.014 25769191

[105] Samimi M Le Gouge A Boralevi F Passeron T Pascal F Bernard P. Topical rapamycin versus betamethasone dipropionate ointment for treating oral erosive lichen planus: a randomized, double-blind, controlled study. J Eur Acad Dermatol Venereol. (2020) 34:2384–91. 10.1111/jdv.16324 32128907

[106] Soria A Agbo-Godeau S Taïeb A Francès C. Treatment of refractory oral erosive lichen planus with topical rapamycin: 7 cases. Dermatology. (2009) 218:22–5. 10.1159/000172830 18997452

[107] Casabona F Gambelli I Casabona F Santi P Santori G Baldelli IA-O. Autologous platelet-rich plasma (PRP) in chronic penile lichen sclerosus: the impact on tissue repair and patient quality of life. Int Urol Nephrol. (2017) 49:573–80. 10.1007/s11255-017-1523-0 28161837

[108] Bolanča Ž Goren A Getaldić-Švarc B Vučić M Šitum M. Platelet-rich plasma as a novel treatment for lichen planopillaris. Dermatol Ther. (2016) 29:233–5. 10.1111/dth.12343 26988129

[109] Pietrzak WS Eppley BL. Platelet rich plasma: biology and new technology. J Craniofac Surg. (2005) 16:1043–54. 10.1097/01.scs.0000186454.07097.bf 16327552

[110] Dhillon RS Schwarz EM Maloney MD. Platelet-rich plasma therapy—future or trend? Arthritis Res Ther. (2012) 14:219. 10.1186/ar3914 22894643 PMC3580559

[111] Knezevic NN Candido KD Desai R Kaye AD. Is platelet-rich plasma a future therapy in pain management? Med Clin North Am. (2016) 100:199–217. 10.1016/j.mcna.2015.08.014 26614728

[112] Jain V Triveni MG Kumar AT Mehta DS. Role of platelet-rich-fibrin in enhancing palatal wound healing after free graft. Contemp Clin Dent. (2012) 3:S240–243. 10.4103/0976-237X.101105 23230372 PMC3514941

[113] Bennardo FA-O Liborio F Barone SA-O Antonelli AA-O Buffone CA-O Fortunato LA-O. Efficacy of platelet-rich fibrin compared with triamcinolone acetonide as injective therapy in the treatment of symptomatic oral lichen planus: a pilot study. Clin Oral Investig. (2021) 25:3747–55. 10.1007/s00784-020-03702-w 33415379

[114] Sethi Ahuja U Puri N More CB Gupta R Gupta D. Comparative evaluation of effectiveness of autologous platelet rich plasma and intralesional corticosteroids in the management of erosive oral lichen planus-a clinical study. J Oral Biol Craniofac Res. (2020) 10:714–8. 10.1016/j.jobcr.2020.09.008 33088702 PMC7557883

[115] Hijazi AA-O Ahmed W Gaafar S. Efficacy of intralesional injections of platelet-rich plasma in patients with oral lichen planus: a pilot randomized clinical trial. Clin Exp Dent Res. (2022) 8:707–14. 10.1002/cre2.550 35218680 PMC9209796

[116] Saglam EA-O Ozsagir ZB Unver T Alinca SA-O Toprak A Tunali M. Efficacy of injectable platelet-rich fibrin in the erosive oral lichen planus: a split-mouth, randomized, controlled clinical trial. J Appl Oral Sci. (2021) 29:e20210180. 10.1590/1678-7757-2021-0180 34614123 PMC8523099

[117] ElGhareeb MA-O Ghoneimy S Elsayed A. Intralesional injection of platelet-rich plasma versus steroid in the treatment of oral lichen planus. J Cosmet Dermatol. (2023) 22:1481–7. 10.1111/jocd.15622 36718838

[118] Al-Hallak N Hamadah OA-O Mouhamad M Kujan OA-O. Efficacy of injectable platelet-rich fibrin in the treatment of symptomatic oral lichen planus. Oral Dis. (2023) 29:2256–64. 10.1111/odi.14261 35593522

[119] Sibani SA McCarron PA Woolfson AD Donnelly RF. Photosensitiser delivery for photodynamic therapy. Part 2: systemic carrier platforms. Expert Opin Drug Deliv. (2008) 5:1241–54. 10.1517/17425240802444673 18976134

[120] Rkein AM Ozog DM. Photodynamic therapy. Dermatol Clin. (2013) 32:415–25. 10.1016/j.det.2014.03.009 24891062

[121] Sharman WM Allen CM van Lier JE. Role of activated oxygen species in photodynamic therapy. Methods Enzymol. (2000) 319:376–400. 10.1016/S0076-6879(00)19037-8 10907528

[122] Edström DW Porwit A Ros AM. Photodynamic therapy with topical 5-aminolevulinic acid for mycosis fungoides: clinical and histological response. Acta Derm Venereol. (2001) 81:184–8. 10.1080/000155501750376276 11558874

[123] Ibbotson SH. An overview of topical photodynamic therapy in dermatology. Photodiagnosis Photodyn Ther. (2010) 7:16–23. 10.1016/j.pdpdt.2009.12.001 20230989

[124] Saleh W Tageldin S Khashaba E Darwish M Elnagdy S Khashaba O. Could photodynamic therapy be utilized as a treatment modality for oral lichen planus? Photodiagnosis Photodyn Ther. (2020) 30:101677. 10.1016/j.pdpdt.2020.101677 32006650

[125] Sulewska MA-O Tomaszuk J Sajewicz EA-O Pietruski J Starzyńska AA-O Pietruska M. Treatment of reticular oral lichen planus with photodynamic therapy: a case series. J Clin Med. (2023) 12:875. 10.3390/jcm12030875 36769523 PMC9917588

[126] Cosgarea R Pollmann R Sharif J Schmidt T Stein R Bodea A. Photodynamic therapy in oral lichen planus: a prospective case-controlled pilot study. Sci Rep. (2020) 10:1667. 10.1038/s41598-020-58548-9 32015380 PMC6997407

[127] Trakarnboonkij J Tanya SA-O Sarideechaigul W Subarnbhesaj A Tabbon P Sattayut SA-O. Case report: recalcitrant oral lichen planus involving bilaterally buccal mucosae treated with a combination of photodynamic and photobiomodulation therapies. F1000Res. (2024) 13:152. 10.12688/f1000research.146733.1 38854440 PMC11162524

[128] Zborowski JA-O Kida DA-O Szarwaryn A Nartowski KA-O Rak P Jurczyszyn KA-O. A comparison of clinical efficiency of photodynamic therapy and topical corticosteroid in treatment of oral lichen planus: a split-mouth randomised controlled study. J Clin Med. (2021) 10:3673. 10.3390/jcm10163673 34441967 PMC8397092

[129] Ozog DM Rkein AM Fabi SG Gold MH Goldman MP Lowe NJ. Photodynamic therapy: a clinical consensus guide. Dermatol Surg. (2016) 42:804–27. 10.1097/DSS.0000000000000800 27336945

[130] Wickenheisser VA Zywot EM Rabjohns EM Lee HH Lawrence DS Tarrant TK. Laser light therapy in inflammatory, musculoskeletal, and autoimmune disease. Curr Allergy Asthma Rep. (2019) 19:37. 10.1007/s11882-019-0869-z 31267251 PMC7357616

[131] Hamblin MR. Mechanisms and mitochondrial redox signaling in photobiomodulation. Photochem Photobiol. (2018) 94:199–212. 10.1111/php.12864 29164625 PMC5844808

[132] Mutafchieva MZ Draganova MN Zagorchev PI Hanna R Tomov GT. Molecular evidence for the efficacy of PBM therapy in the treatment of oral lichen planus. Photodiagnosis Photodyn Ther. (2025) 51:104479. 10.1016/j.pdpdt.2025.104479 39798779

[133] Mutafchieva MA-O Draganova MA-O Yaneva BA-O Zagorchev PA-O Tomov GA-O. Clinical improvement and P63-deficiency correction in OLP patients after photobiomodulation. Dent J (Basel). (2024) 12:338. 10.3390/dj12110338 39590388 PMC11593062

[134] Pires de Sousa MV Ferraresi C Kawakubo M Kaippert B Yoshimura EM Hamblin MR. Errata: transcranial low-level laser therapy (810 nm) temporarily inhibits peripheral nociception: photoneuromodulation of glutamate receptors, prostatic acid phophatase, and adenosine triphosphate. Neurophotonics. (2016) 3:019801. 10.1117/1.NPh.3.1.019801 26835486 PMC4725212

[135] Leyane TS Jere SW Houreld NA-O. Cellular signalling and photobiomodulation in chronic wound repair. Int J Mol Sci. (2021) 22:11223. 10.3390/ijms222011223 34681882 PMC8537491

[136] Shamloo S Defensor E Ciari P Ogawa G Vidano L Lin JS. The anti-inflammatory effects of photobiomodulation are mediated by cytokines: evidence from a mouse model of inflammation. Front Neurosci. (2023) 17:1150156. 10.3389/fnins.2023.1150156 37090796 PMC10115964

[137] Mohamed RK Elsayed NM Mahmoud SA Gaweesh YY. Photobiomodulation versus corticosteroid in the management of erosive oral lichen planus: a randomized controlled clinical trial. BMC Oral Health. (2024) 24:246. 10.1186/s12903-024-03976-6 38365694 PMC10873933

[138] Sobral SS da Silva Brandão EH de Barros Gallo C Molon A Sobral APT de Fátima Teixeira da Silva D. Analysis of the psychopathological profile, quality of life, and cost-effectiveness of oral lichen planus patients treated with photobiomodulation. Clin Oral Investig. (2022) 26:719–28. 10.1007/s00784-021-04050-z 34251533

[139] Nammour SA-O El Mobadder MA-O Brugnera AA-OX Namour MA-O Houeis S Heysselaer D. Photobiomodulation therapy vs. Corticosteroid for the management of erosive/ulcerative and painful oral lichen planus. Assessment of success rate during one-year follow-up: a retrospective study. Healthcare (Basel). (2021) 9:1137. 10.3390/healthcare9091137 34574912 PMC8466159

[140] Roccon AA-O Cavallin FA-O Zanette GA-O Bacci CA-O. Single session of laser photobiomodulation for symptom management of oral lichen planus: a retrospective study. Lasers Med Sci. (2023) 38:43. 10.1007/s10103-023-03706-4 36656450 PMC9849837

[141] Ioannides DA-O Vakirlis E Kemeny L Marinovic B Massone C Murphy R. European S1 guidelines on the management of lichen planus: a cooperation of the European dermatology forum with the European academy of dermatology and venereology. J Eur Acad Dermatol Venereol. (2020) 34:1403–14. 10.1111/jdv.16464 32678513

[142] Li K Hua H He W. Characteristics of the psychopathological status of oral lichen planus: a systematic review and meta-analysis. Aust Dent J. (2022) 67:113–24. 10.1111/adj.12896 35067951

[143] Vallejo MJ Huerta G Cerero R Seoane JM. Anxiety and depression as risk factors for oral lichen planus. Dermatology. (2001) 203:303–7. 10.1159/000051777 11752817

[144] Pippi R Romeo U Santoro M Del-Vecchio A Scully C Petti S. Psychological disorders and oral lichen planus: matched case-control study and literature review. Oral Dis. (2016) 22:226–34. 10.1111/odi.12423 26680999

[145] Mehdipour M Taghavi-Zenouz A Farnam A Attaran R Farhang S Safarnavadeh M. The relationship between anger expression and its indices and oral lichen planus. Chonnam Med J. (2016) 52:112–6. 10.4068/cmj.2016.52.2.112 27231675 PMC4880575

[146] Glavina A Zoranić A Tadin A Cigić L Šupe-Domić D Lugović-Mihić L. Is salivary *Α*-amylase a reliable indicator of psychological Status and quality of life in patients with oral lichen planus: a case-control study. Cell Physiol Biochem. (2024) 58:311–21. 10.33594/000000714 39012386

[147] Liao H Luo Y Long L Peng J Qiu X Yuan P. Anxiety and oral lichen planus. Oral Dis. (2021) 27:506–14. 10.1111/odi.13569 32697012

[148] Radwan-Oczko M Zwyrtek E Owczarek JE Szcześniak D. Psychopathological profile and quality of life of patients with oral lichen planus. J Appl Oral Sci. (2018) 18:e20170146. 10.1590/1678-7757-2017-0146 PMC5777404 29364344

